# Large‐scale heavy precipitation over central Europe and the role of atmospheric cyclone track types

**DOI:** 10.1002/joc.5386

**Published:** 2017-12-21

**Authors:** Michael Hofstätter, Annemarie Lexer, Markus Homann, Günter Blöschl

**Affiliations:** ^1^ Department of Climate Research Central Institute for Meteorology and Geodynamics Vienna Austria; ^2^ Institute of Geography, Physical Geography and Quantitative Methods, University of Augsburg Germany; ^3^ Institute of Hydraulic Engineering and Water Resources Management, Vienna University of Technology Austria

**Keywords:** synoptic climatology, precipitation, cyclones, cyclone track, atmospheric circulation, heavy precipitation, climate change

## Abstract

Precipitation patterns over Europe are largely controlled by atmospheric cyclones embedded in the general circulation of the mid‐latitudes. This study evaluates the climatologic features of precipitation for selected regions in central Europe with respect to cyclone track types for 1959–2015, focusing on large‐scale heavy precipitation.

The analysis suggests that each of the cyclone track types is connected to a specific pattern of the upper level atmospheric flow, usually characterized by a major trough located over Europe. A dominant upper level cut‐off low (COL) is found over Europe for strong continental (CON) and van Bebber's type (Vb) cyclones which move from the east and southeast into central Europe. Strong Vb cyclones revealed the longest residence times, mainly due to circular propagation paths.

The central European cyclone precipitation climate can largely be explained by seasonal track‐type frequency and cyclone intensity; however, additional factors are needed to explain a secondary precipitation maximum in early autumn. The occurrence of large precipitation totals for track events is strongly related to the track type and the region, with the highest value of 45% of all Vb cyclones connected to heavy precipitation in summer over the Czech Republic and eastern Austria. In western Germany, Atlantic winter cyclones are most relevant for heavy precipitation. The analysis of the top 50 precipitation events revealed an outstanding heavy precipitation period from 2006 to 2011 in the Czech Republic, but no gradual long‐term change. The findings help better understand spatio‐temporal variability of heavy precipitation in the context of floods and may be used for evaluating climate models.

## 1 Introduction

Precipitation is a key element of the earth's climate system through the vertical and horizontal transport of mass and energy (Trenberth and Stepaniak, 2004; Hantel, 2005; L'Ecuyer *et al*., 2015). The long‐term characteristics of precipitation vary substantially between regions (Peel *et al*., 2004; Kottek *et al*., 2006; Rubel and Kottek, 2010) due to latitudinal difference and local geographical features such as proximity to large open water bodies and different land‐surface properties, even within a rather small domain as Europe (Haylock *et al*., 2008). Besides regional differences in precipitation characteristics, seasonal patterns exist (Zveryaev, 2004, 2006) which are typically caused by annual variations of air temperatures, solar insolation, dominant atmospheric weather patterns and vegetation dynamics. On top of that, internal climate variability induces large variations of dry or wet conditions at decadal and inter‐annual timescales (Rimbu *et al*., 2001; Schmidli *et al*., 2002; Casty *et al*., 2005; Pauling *et al*., 2006; Masson and Frei, 2016; Haslinger and Blöschl, 2017). Hydro‐meteorological extremes are of particular relevance. Extreme precipitation has caused several devastating floods in central Europe over the last 20 years. Examples include the July 1997 flood in Poland (Kundzewicz *et al*., 1999), Germany, the Czech Republic and Austria; the May 1999 flood in Bavaria and western Austria (Bayrisches Landesamt für Wasserwirtschaft, BLfW (Hrsg.), 2003); the August 2002 flood hitting several central European countries (e.g. Grazzini and van der Grijn, 2002); the August 2005 flood in the German/Austrian Alpine Region (Bayerisches Landesamt für Umwelt (BLU), 2006); the June 2006 and May/June as well as August 2010 floods in the Czech Republic (Müller *et al*., 2015); the June/July 2009 flood in central and eastern Europe (Danhelka and Kubát, 2009); the June 2013 flood in Bavaria and Austria (Blöschl *et al*., 2013) and the May 2014 flood on the Balkans and in southeast Europe (Stadtherr *et al*., 2016). A potential increase of the frequency or intensity of hydro‐meteorological wet events is of great concern in the ongoing climate change debate (Trenberth *et al*., 2003; Westra *et al*., 2014). Such events tend to result from anomalous atmospheric circulation patterns over Europe (e.g. Grams *et al*., 2014; Kašpar and Müller, 2014; Dayan *et al*., 2015) in combination with specific hydrologic boundary conditions on the surface such as antecedent soil moisture and soil infiltration capacity (e.g. Hall *et al*., 2014; Nied *et al*., 2014). Additionally, seasonality is a very important factor both in terms of the atmospheric circulation as the driving mechanism (Zveryaev and Allan, 2010; Casanueva *et al*., 2014; Fleig *et al*., 2015; Scherrer *et al*., 2016) and for the resulting flood processes in terms of soil moisture, snow processes and frozen ground (Merz and Blöschl, 2003; Parajka *et al*., 2010; Müller *et al*., 2015; Blöschl *et al*., 2017). An increasing number of studies have focused on the specific characteristics of the atmospheric circulation during heavy precipitation events in recent years. For example, two circulation types have been isolated in for the heaviest Czech Republic rainfall events during 1958–2002 (Müller *et al*., 2009). The first circulation type consists of a major, quasi‐stationary trough located over Europe, steering a number of fast moving, frontal short waves over the Czech territory. In contrast, the second type is characterized by a COL over central Europe, with a cyclone at lower atmospheric levels moving very slowly. This is a similar situation to those of the four historic flood events in Austria/Bavaria in September 1899, July 1954, August 2002 and May 2013, where a major upper level COL was observed over central Europe, accompanied by cyclones located at the surface level around the Eastern Alps (Blöschl *et al*., 2013). For Switzerland, stationarity of anomalous circulation patterns was a key issue for the top 24 floods between 1868 and 2005, leading to repeated periods of heavy precipitation over a few days (Stucki *et al*., 2012). For the Elbe river basin, a specific circulation anomaly (which the authors termed pattern 29) could be identified as producing long‐duration rain episodes and hence floods, irrespective of the initial soil moisture (Nied *et al*., 2014). This circulation anomaly is characterized by an upper level COL over the Gulf of Genoa and was found to be more frequent in summer than in winter.

Selected outstanding events (May 2014, June 2013 and August 2002) have been investigated in very much detail by a number of authors (Ulbrich *et al*., 2003b; Grams *et al*., 2014; Stadtherr *et al*., 2016). For these cases, a quasi‐persistent upper level COL induced a series of surface cyclones which propagated in anti‐clockwise direction around the respective regions hit by heavy precipitation. Cyclones propagating from the region of Genoa into central Europe are usually referred to as following a Vb track after van Bebber (1891). This cyclone type has been investigated systematically in recent years. Nissen *et al*. (2013) found that 40% of Vb cyclones are related to heavy precipitation (95th percentile) in the Elbe catchment, with about two thirds of these events occurring in April/May. Similarly, Messmer *et al*. (2015) revealed 30% of the summer Vb cyclones exceed the 95th precipitation percentile in the Alpine region (2% in winter).

While all of these studies have highlighted the importance of specific cyclone tracks in producing heavy precipitation in central Europe, the spatio‐temporal characteristics of large‐scale precipitation have so far not been related to cyclone tracks in a systematic way. The aim of this study therefore is:

1. To explore the climatologic characteristics of heavy and mean precipitation, observed in the vicinity of atmospheric cyclones, for selected regions in central Europe and relate them to cyclone track types.
2. To analyse the climatological characteristics of cyclone track types in this regard.
3. To identify dominant circulation patterns over Europe in the context of cyclone track types.

The findings are intended to foster the understanding of large‐scale heavy precipitation and related flood events from the perspective of the atmospheric drivers. They are also intended to provide a basis for evaluating climate models over Europe, e.g. by comparing modelled frequency, intensity, seasonality and temporal variability of each cyclone track type with observations.

## 2 Data and analysis procedure

### 2.1 Cyclone track types

**Figure 1:**
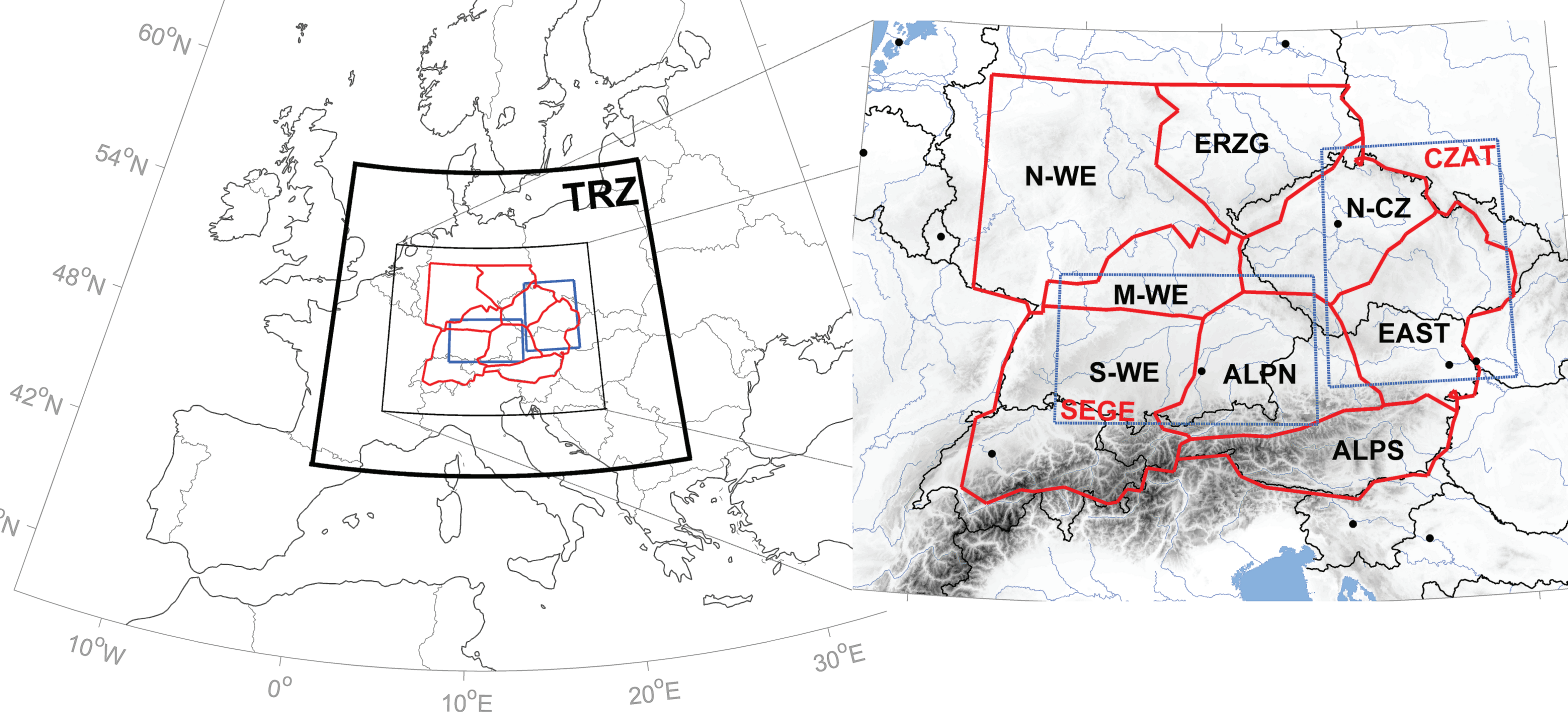
Study region located within the TRZ (black bold line) and subregions used for precipitation analysis shown in red for 1959–2006 (WETRAX data) and blue for 1959–2015 (E‐OBS data).

The cyclone track types used in this study were identified by the classification scheme of Hofstätter *et al*. (2016) developed for the European domain. The tracking scheme has been developed by Murray and Simmonds (1991) and Simmonds *et al*. (1999). In addition, it considers both open and closed systems (Pinto *et al*., 2005) and allows for splitting and merging of cyclone tracks. Closed systems are localized by finding local pressure minima whereas open systems are localized by identifying local vorticity maxima within open troughs. Cyclones are first identified at time *t*_*n*_, then a first guess for the position at time *t*_*n+*1_ is calculated and finally tracks are identified by scoring the direction and distance between the first guess and all candidate cyclones at time *t*_*n+*1_. For the tracking, geopotential height at the atmospheric level of 700 hPa (Z700) and air pressure at sea surface level (SLP) are used separately. Cyclones are tracked within a domain ranging from 40°W to 50°E at 65°N and 20°W to 40°E at 30°N. In order to avoid entrance/exit problems, splitting and merging are refused near the margin. Weak cyclones and spurious tracks are excluded from the analysis (Hofstätter *et al*., 2016). In the current study, the cyclone track analysis is based on the Japanese 55‐year reanalysis (JRA‐55) of the Japanese Meteorological Agency (Kobayashi *et al*., 2015; Harada *et al*., 2016) retrieved from the Research Data Archive at the National Center for Atmospheric Research (NCAR) (Japanese Meteorological Agency, 2013) at 1.25° spatial resolution. JRA‐55 is a regularly updated global atmospheric reanalysis at 6 hourly temporal resolution, extending back to 1958 and has been used instead of ERA‐40 (Uppala *et al*., 2005) to cover the most recent years. In order to exclude small‐scale or spurious cyclones, especially over mountain orography, the data are filtered by a discrete spatial low‐pass filter (Freser and von Storch, 2005) that removes any structures smaller than 400 km, relaxes smoothly up to 1000 km and lets all large scales pass through (Hofstätter and Chimani, 2012). Cyclone track and precipitation characteristics only refer to those cyclones that are located within a ‘track recognition zone’ (TRZ, 0.5°–23.9°E and 42.3°–56.2°N) for at least 18 h (Figure 1, left). For all other cyclones passing outside of TRZ, a track type cannot be assessed; hence, such cyclones were not considered in this study. All cyclone tracks, identified either at Z700 or at SLP, were pooled into one sample denoted as ‘Z700/SLP’. In specific instances they were treated separately which is specifically indicated in this article.

**Figure 2:**
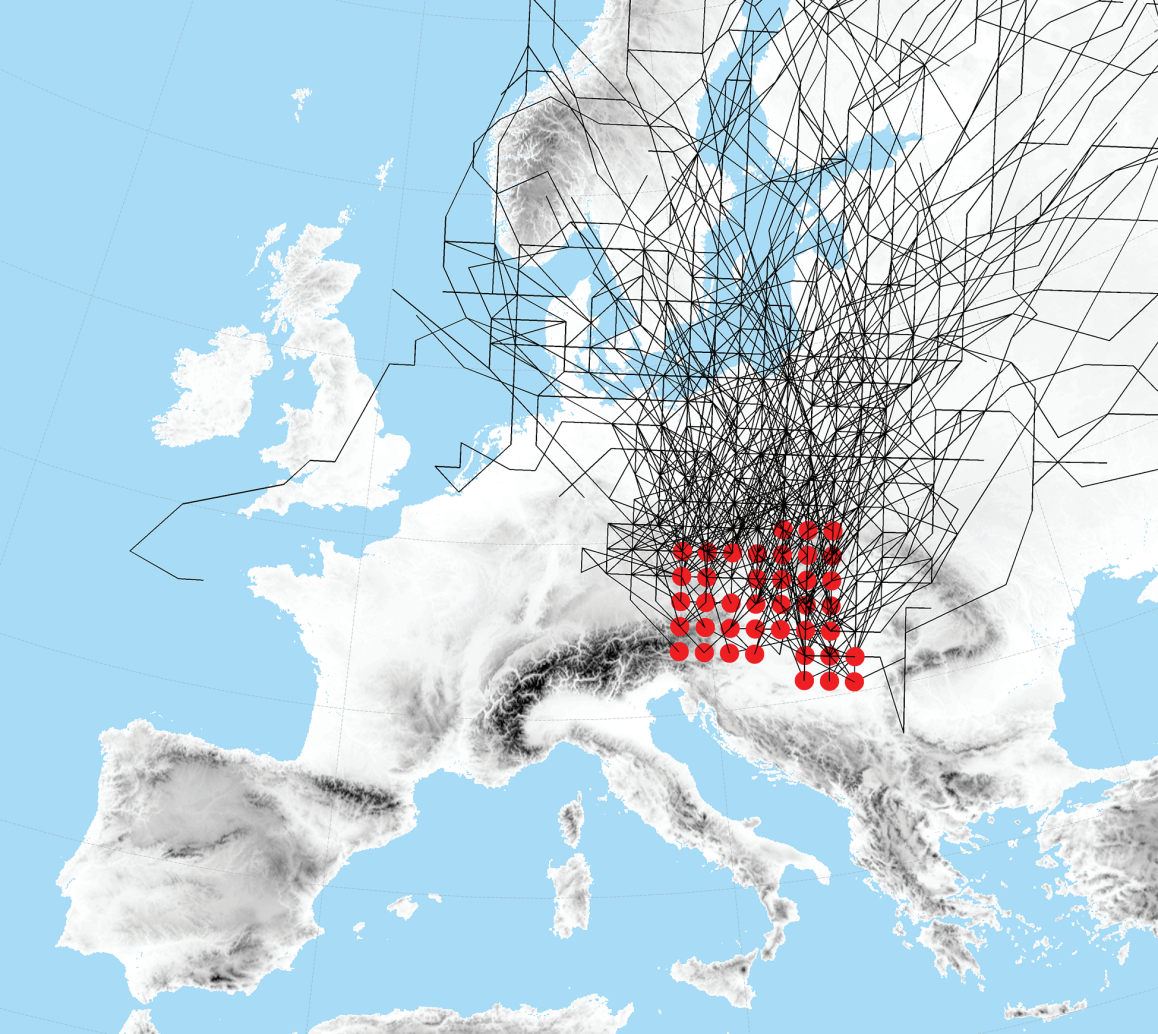
Members of the cyclone track type ‘EA’ (SLP and Z700, 1959–2015). Red dots indicate the location of first detection of individual tracks.

The classification of Hofstätter et al. (2016) consists of nine different types, based on the geographic regions the cyclones traverse before entering central Europe (Table 1). In this study an additional track type ‘Eastern Alpine’ (EA) was considered which represents cyclones propagating to the northeast over eastern Europe, similar to type Vb (van Bebber, 1891), but developing on the eastern leeside of the European or Dinaric Alps (Figure 2). Cyclone tracks of type EA were previously a subset of type TRZ but were classified separately here because of their peculiarity in terms of propagation and related precipitation characteristics, which have emerged in the course of this study. Cyclone tracks were determined for the years 1959–2015. As a measure for cyclone intensity, relative geostrophic vorticity rv was used in this study. In order to make cyclones between different levels comparable, rv was log‐transformed by ln(rv_Z700_) and ln(0.7*rv_SLP_), and used as a score variable.

**Table 1:** Cyclone track types of Hofstätter et al. (2016) plus the additional type EA.

Number	Acronym	Type
1	Vb	van Bebber's type ‘five‐b’
2	EA	Eastern Alpine track
3	X‐N	Northwards propagation, emerging from the northern Adriatic Sea or Mediterranean Sea
4	X‐S	Southwards propagation, emerging from the northern Adriatic Sea or Mediterranean Sea
5	MED	Mediterranean
6	STR	Subtropical
7	ATL	Atlantic
8	POL	Polar
9	CON	Continental
10	TRZ	All tracks emerging within TRZ (except Vb, X‐N, X‐S)

### 2.2 Precipitation data and study region

The study region for precipitation analysis is located over central Europe within region TRZ, covering Austria as well as large parts of Germany, Switzerland and the Czech Republic (Figure 1, red and blue). Two gridded time series of daily precipitation were used in this study: the WETRAX data at 6 km resolution covering the period 1959–2006 (Hofstätter *et al*., 2015) and the E‐OBS data (v13.1) at 20 km resolution, retrieved from the ECA&D data portal, covering the period 1959–2015 (Haylock *et al*., 2008). The WETRAX data are based on station data from the Austrian Weather Service (ZAMG) for the Austrian territory and for the remaining parts of the study region from the German Weather Service (DWD – the HYRAS data set; Rauthe *et al*., 2013). Because the motivation for this study was to understand the relationship between atmospheric cyclone tracks and large‐scale precipitation patterns over central Europe, the WETRAX precipitation data were aggregated over regions deemed to exhibit similar spatio‐temporal variability (red lines in Figure 1). These regions were delineated by S‐mode (Richman, 1986), orthogonally varimax‐rotated principal component analysis (PCA) on the gridded daily data. The principal components (PCs) are based on the correlation matrix of the input variables. The highest loadings of each PC were used to define regions of similar precipitation variability. The loadings are the correlation coefficients between the variables and the principle components. The number of PCs to be extracted was determined by the dominance criterion (Jacobeit, 1993) resulting in eight regions (Figure 1, red lines), which have an explained variance of *R* ^2^ = 0.67 on an annual basis.

The E‐OBS data were used for a complementary analysis over two selected regions, southeastern Germany (SEGE) and CZAT (Figure 1, blue lines), for the extended period 1959–2015. E‐OBS is heavily affected by smoothing of large‐scale extremes (Hofstra *et al*., 2010) as well as by a strong underestimation of smaller‐scale events (Zolina *et al*., 2014) in regions with low station densities, particularly in the convective summer season. Station density is about 350 km^−2^ in Germany and about 600 km^−2^ in the Czech Republic, but much lower in the Austrian Alps. As the low density affects small parts of SEGE and CZAT regions, the extended analysis only considers highly ranked large‐scale precipitation events in these regions. The WETRAX data set, in contrast, provides a comparably high station density over Germany (∼100 km^−2^) and Austria (∼140 km^−2^) as well as a similar density as E‐OBS over the Czech Republic (∼550 km^−2^). Precipitation events being smaller than about 50 × 50 km are therefore not well resolved in the N‐CZ region and in the northern parts of the EAST region. Because this study focuses on large‐scale precipitation observed in the vicinity of cyclones, convective precipitation is much less relevant.

The cyclone track data are available four times a day at 0000, 0006, 0012 and 0018 UTC in contrast to the daily precipitation data, which are reported once a day at 0006 UTC and refer to the subsequent 24 h time frame. In order to associate precipitation with cyclone tracks, regional averages of daily precipitation totals (dREF) were interpolated to 6 hourly values following the cyclone dates. A fourth of the daily precipitation was allocated to 0018 UTC of the corresponding date and the missing values at 0000, 0006 and 0012 UTC were estimated by applying a piecewise cubic spline. In order to evaluate the effect of interpolation, the 6 hourly data were re‐aggregated into daily totals (dINT) and compared to dREF. Although the interpolation allows for considering information from adjacent time steps, it also leads to smoothing effects, with an overestimation of the total precipitation amount for dREF < 5 mm day^−1^ by about +17% and an underestimation for dREF ≥ 5 mm day^−1^ by about –8%, with some variations between regions. Therefore, a quantile‐mapping (QM) bias correction (Panofsky and Brier, 1968) was applied by estimating a transfer function based on the difference between the sorted values of dINT and dREF (Themeßl *et al*., 2011). The transfer function was approximated by first fitting a sinusoidal function (sin2), followed by fitting a Gaussian (gaus2) function to the residuals. The sum of both functions was applied as the transfer function to the 6 hourly precipitation according to the corresponding quantiles. As a result, the bias could be reduced to about +5% for small and to about −1% for high precipitation amounts. Although QM is able to adjust all statistical moments, it does not account for any changes in the temporal structure arising from interpolation. In the case of low precipitation amounts (dREF < 1 mm), the correlation between dREF and dINT drops to *R* ^2^ = 0.45 for the sqrt‐transformed daily data. For larger precipitation amounts, temporal correlation is preserved (*R* ^2^ = 0.95), particularly for heavy precipitation (dREF > 95%) with *R* ^2^ ≥ 0.96 for all regions.

### 2.3 Analysis of mean and heavy precipitation

An overview of the analysis methods, as detailed below, is shown in Table 2. For the climatologic analysis of mean and heavy cyclone precipitation, 6 hourly precipitation (*R* ^6^) was assigned to a cyclone track if that cyclone was located within region TRZ at that time. The size of region TRZ is about 1600 × 1600 km which was chosen in accordance with the mean radius of mid‐latitude cyclones, which is between 350 and 800 km, depending on the cyclones' life stage (Wernli and Schwierz, 2006). In case more than one cyclone was present at the same time, precipitation was assigned to every candidate cyclone, as a unique attribution would require a manual analysis of the specific synoptic situation. At SLP this is the case for 23% of the cyclones as compared to Z700 with just 15%.

**Table 2:** Precipitation measures used in this study.

Measure	*R* ^6^	*R* ^trc^	*R* ^24^
Description	Regional average at time *t*	Cyclone track precipitation	24 h running total at time *t*
Calculation	Interpolated values at *t* = 0000, 0006, 0012 and 0018 UTC from daily rain gauge measurements	Sum of *R* ^6^ for the duration *T* of a cyclone track in TRZ, $\sum_{t=1}^{t=T}R_{\left(t\right)}^{6}$	$\frac{\left(R_{\left(t-12\right)}^{6}+R_{\left(t+12\right)}^{6}\right)}{2}+\sum_{t-6}^{t+6}R_{\left(t\right)}^{6}$
Used for the analysis of	Mean cyclone precipitation (chap. 3.2.a)	Heavy cyclone precipitation (chap. 3.2.b)	Top 50 precipitation events (chap. 3.2.c)
Data base	WETRAX (1959–2006)		WETRAX (1959–2006), E‐OBS (1959–2015)
Drawback	–	Depends on *R* ^6^ and on residence time rt	Restricted to 24 h, but longer and shorter intervals may also be relevant for floods

The total precipitation amount of a certain cyclone, denoted as *R* ^trc^, was determined by summing up precipitation (*R* ^6^) as long as a cyclone was within TRZ. *R* ^trc^ is used for the heavy precipitation assessment and depends on both the magnitude of *R* ^6^ and the residence time *rt* of an individual cyclone (Table 2, centre). In this study heavy precipitation is defined as precipitation exceeding 95th percentile of *R* ^trc^ which is termed HP_95_. In order to estimate HP_95_ robustly, the distribution of the largest values of *R* ^trc^ was fitted by a generalized Pareto distribution *G*(*R* ^trc^)

(1) GRtrc=1−1+Rtrc·ξβ−1/ξξ≠01−e−Rtrc/βξ=0

(2) $$n=\text{round}\;\left(15+N/10\right)$$

where *β* (scale parameter) and *ξ* (shape parameter) were estimated by the maximum‐likelihood method (Coles, 2001), *n* is the number of largest values from *N* values of *R* ^trc^. Equation (2) assures a sufficient sample size *n*, in case of track types with a low number of cyclones. The values in Equation (2) were chosen subjectively by examining the sample mean excess function in combination with a Hill plot for different track types on a seasonal basis. For example, in case of Vb summer (May–October) cyclones with *N* = 120, the Hill estimator stabilizes at *n* = 25 and the mean excess function is nonlinear above a threshold of 33 mm or *n* < 24. When using Equation (2) this leads to a computational threshold for fitting the generalized Pareto distribution of *n* = 27. As a consequence, *n* always corresponds to at least 10% of the largest values of *N* and increases for low values of *N*.

### 2.4 Analysis of outstanding precipitation events

In order to further explore individual precipitation events, the 50 largest running 24 h precipitation totals (*R* ^24^) in the period 1959–2006 as well as in the extended period 1959–2015 were identified. *R* ^24^ was calculated by a centred moving average using weights of 0.5–1–1–1–0.5 on consecutive 6‐hourly values (Table 2, right). There is an important difference between *R* ^trc^ and *R* ^24^ as the latter does not depend on cyclone residence time. Next a weighted cyclone intensity *rdv* ^*∼*^ is calculated (Equations (3) and (4)) for each cyclone, based on the relative geostrophic vorticity rv and the distance dis between the cyclone centre and the centre of the respective study region.

(3) $$\mathrm{rdv}=\left\{\begin{array}{c} {\mathrm{rv}}^{*}{\left(1-\left[\frac{{\mathrm{dis}}^{2}}{{d_{\max}}^{2}}\right]\right)}^{2}\;\left(0<\mathrm{dis}\leq d_{\max}\right)\\ 0\;\left(\text{otherwise}\right) \end{array}\right.$$

(4) $${\mathrm{rdv}}^{∼}=\text{mean}\;\left({\mathrm{rdv}}_{\left(t-6\right)};{\mathrm{rdv}}_{\left(t\right)}\right)$$

By this approach, cyclones are more likely attributed to a top 50 events at time *t* if they are strong and/or if they pass by very close to the respective region. All cyclones located outside a distance of *d*_max_ = 1500 km were disregarded because typical cyclone extents are far smaller than *d*_max_ (Schneidereit *et al*., 2010). Only the strongest/closest cyclone per track type *τ* was considered at each level, using

(5) $${\mathrm{rdv}}_{Z7,\mathrm{SLP}}^{\max\left(\tau \right)}=\max{\left({\mathrm{rdv}}_{\mathrm{Vb}}^{∼},\ldots ,\;{\mathrm{rdv}}_{\mathrm{TRZ}}^{∼}\right)}_{Z7,\mathrm{SLP}}$$

Finally, the cyclone intensity for each track type was calculated by summing the cyclone intensities over both levels using ${\mathrm{rdv}}^{\max\left(\tau \right)}={\mathrm{rdv}}_{\mathrm{SLP}}^{\max\left(\tau \right)}+{\mathrm{rdv}}_{Z7}^{\max\left(\tau \right)}$ which was used for the attribution, with higher values indicating a more conclusive attribution. This approach allows to reliably assign track types to the top 50 events, even in complex synoptic situations when several cyclones, and cyclones with different track types, are observed simultaneously.

## 3 Results

### 3.1 Cyclone track types

#### 3.1.1 Characteristics of track types

Track‐type characteristics have been derived for the period 1959–2015 in the following. The mean annual frequency of cyclone track types is presented in Table 3. About 92 cyclones traverse region TRZ per year (108 at SLP; 76 at Z700), in correspondence with Messmer *et al*. (2015) who found 99 cyclones per year over a similar European domain at the level of Z850. The overall number of tracks is about 10% smaller than that of Hofstätter *et al*. (2016) which may be related to the different input data (ERA‐40 in their case, and JRA‐55 in this article) as different reanalysis data usually result in different cyclone counts (e.g. Tilinina *et al*., 2013). Apart from resolution issues (Hodges *et al*., 2011), this effect tends to be strongest for weak, slow moving cyclones (Raible *et al*., 2008). The relative frequency of the track types, however, is almost identical with the largest contribution coming from type ATL (25%), TRZ (24%), X‐S (17%) and MED (12%), which together accounting for nearly 80% of the cyclones. The other cylones were allocated to types X‐N, POL and Vb, as well as EA, STR and CON, with only one event per year found for the least frequent type STR. The ratio of cyclone tracks between SLP and Z700 is 1.42 as shown in Table 3. As splitting or merging of cyclones is only slightly more frequent at SLP (22%) than at Z700 (16%), the higher number of tracks at SLP arises from a higher number of individual cyclones found at this level. Neu *et al*. (2013) found a similar ratio of 1.22; however, their study covered the entire Northern Hemisphere. Types STR, EA, X‐S and MED are relatively more frequent at SLP, while CON, Vb, POL and ATL are more frequent at Z700.

**Table 3:** Mean annual number of cyclone tracks in central Europe 1959–2015 as well as the ratio of cyclone tracks between the level SLP and Z700.

	Vb	EA	X‐N	X‐S	MED	STR	ATL	POL	CON	TRZ	ALL
(year)	4.8	1.8	5.1	15.2	10.8	1.0	22.5	6.5	1.9	22.3	91.9
(%)	5.2	2.0	5.5	16.5	11.7	1.1	24.5	7.1	2.1	24.3	100
Ratio SLP/Z700	0.84	2.12	1.70	2.00	1.96	2.93	1.15	0.90	0.51	1.53	1.42

**Figure 3:**
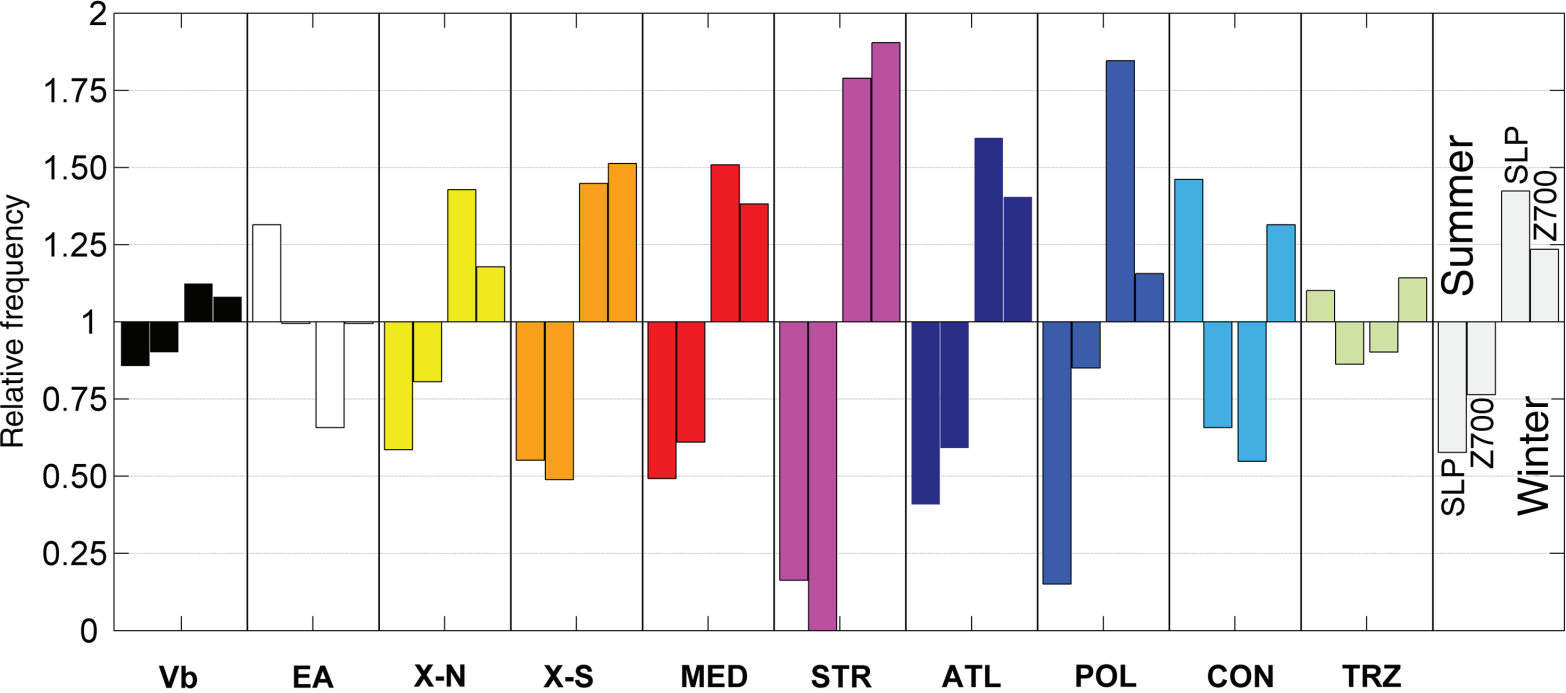
Share of strong cyclones between summer and winter at the level of SLP and Z700 (1959–2015) for each track type (coloured bars) as well as for all tracks (grey bars). A value of one indicates an equal fraction of strong cyclones between winter and summer at a certain level.

The relative frequency of strong cyclones at SLP and Z700 is shown for the summer and winter seasons separately in Figure 3. Summer refers to the period May–October and winter to the period November–April. A value of unit relative frequency indicates that the share of strong cyclones between summer and winter is identical for a given type at a given atmospheric level. Strong cyclones tend to be more frequent in winter than in summer (grey bars in Figure 3), with a larger difference at SLP (1.4 *vs* 0.6) than at Z700 (1.2 *vs* 0.8). This applies to most track types, but strong POL cyclones obviously do not occur at SLP in summer at all, indicating a special cyclone type ‘Kaltlufttropfen’ (Llasat and Puigcerver, 1990) among the track types. Strong STR cyclones only occur in winter, at times when major upper level troughs propagate down to the Iberian Peninsula more frequently. In contrast to the overall characteristics, EA cyclones are twice frequent at SLP than at Z700 (Table 3), as shown before, and strong EA cyclones mostly occur in summer at SLP. For these cases, a pronounced westerly flow is usually found over the Eastern Alps or the Dinaric Alps at upper atmospheric levels, promoting the development of a lower level vortex at the respective mountain's leeside. This is plausible and consistent with synoptic observations. However, the reasons for a reversed seasonality for this type at SLP remain unclear. Almost all of the EA‐systems emerge at the eastern leeside of the Alps or the Dinaric Alps as a unique system and are not a consequence of an interrupted Vb track at SLP, or the successor of a Vb track at Z700, as confirmed by manual checks.

**Figure 4:**
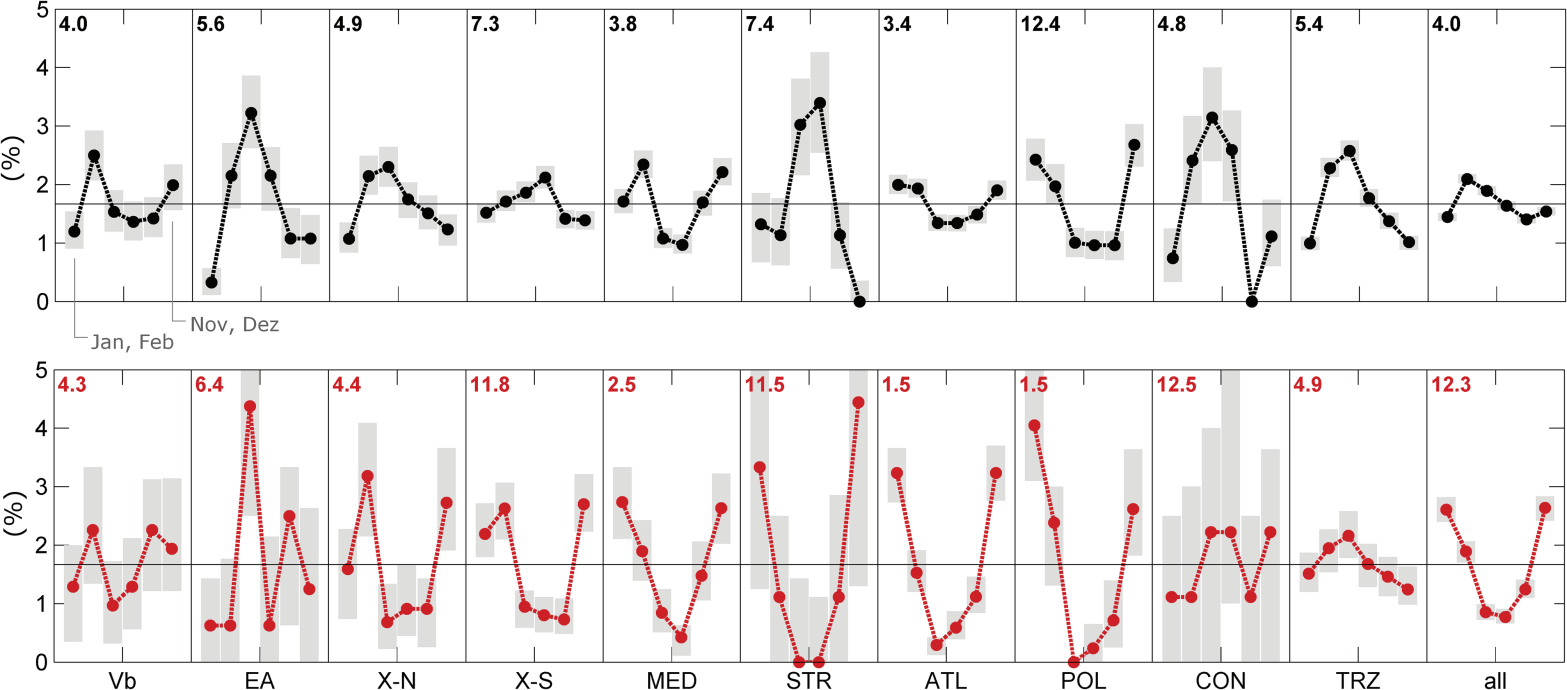
Mean annual cycle of the frequency of track types for bimonthly periods for weak (top, black) and strong (bottom, red) cyclones (1959–2015). Number in the top left corner indicates the frequency peak (month, e.g. 12.5 indicates mid‐December). Grey bars indicate the confidence intervals (α = 0.05).

In order to analyse the seasonal characteristics more in depth, Figure 4 shows the seasonal distribution of the cyclone track frequency together with the frequency peak stated as the calendar month. Grey bars indicate the confidence intervals (α = 0.05) for the mean seasonal cycle which were calculated from 10^4^ bootstrap samples with replacement. For this analysis, cyclone tracks were divided into strong and weak tracks based on the 85th percentile of cyclone intensity as a threshold. Cyclone intensity was quantified as the peak value of the relative geostrophic vorticity rv observed within TRZ as an 18 h running mean. The frequency of strong cyclones (Figure 3, bottom right) exhibits a pronounced seasonal cycle, while this is not the case for the weak cyclones (top right). Weak cyclones are most frequent at the beginning of April, whereas strong cyclones are most frequent during November–March with the frequency peak in mid‐December. Strong TRZ, CON, EA and Vb cyclones may occur in any season. Summer cyclones of the Vb type are particularly strong at SLP – similar to Atlantic winter cyclones – as shown by Hofstätter et al. (2016). A secondary maximum in frequency can be recognized for Vb in late autumn (September–December), at a time when sea surface temperatures are still high in the genesis region around northern Italy. The secondary maximum has also been recognized by Hofstätter and Chimani (2012) for Vb as well as by Flocas (1988) for cyclones developing over the western Mediterranean Sea, northern Italy or the northern Adriatic Sea between 40° and 45°N, which corresponds very well with the Vb source region used in this study.

Another characteristic that is relevant to precipitation is the time a cyclone remains close to the study region. The residence time within region TRZ has been therefore been calculated (Table 4). On average, cyclones remain about 35 h or 1.45 days over central Europe, except for type Vb with nearly 60 h or 2.4 days. For strong cyclones (cyclone intensity >85th percentile) the average is higher with 54 h or 2.25 days, indicating a lower propagation speed for this group. In case of strong Vb cyclones, the residence times are even longer with more than 3 days or 80 h. For all other types the residence time is close to the average. The residence time must be related either to the propagation speed or to the specific path of cyclones within region TRZ. On average, cyclones move at a speed of 8.7 m s^−1^ and for 80% of all cyclones the propagation speed is between 6.5 and 11.8 m s^−1^, with about 10% lower speeds in summer compared to the winter (not shown). The difference between Z700 and SLP is minor but strong cyclones are about 15% slower than all others. This means that strong summer cyclones move at considerably lower speeds over central Europe which explains the difference in residence time. These velocities are very similar to the ones found in other studies, such as velocities in winter for the Northern Hemisphere (Neu *et al*., 2013) or on an annual basis over the western Mediterranean and parts of central Europe (Lionello *et al*., 2016). Stratified by the track type, the fastest and slowest cyclones are found among types ATL and X‐S, respectively. There are also systematic differences between the other types; however, these are not very pronounced and a broad range of speeds occurs.

**Table 4:** Mean duration cyclone centres remain within TRZ (days) 1959–2015.

Residence time	Vb	EA	X‐N	X‐S	MED	STR	ATL	POL	CON	TRZ	ALL
All intensities	2.43	1.39	1.58	1.34	1.32	1.30	1.34	1.40	1.58	1.48	1.45
Strong cyclones	3.50	2.00	2.25	2.00	2.00	2.00	2.00	2.00	2.25	2.25	2.25

As shown above (Table 4), the residence times of Vb are much longer than those of the other types, but this is not reflected by lower propagation speeds. This is because of the typical circular shape of propagation paths within TRZ, steered either by orographic features or by upper level atmospheric circulation patterns favoring quasi‐stationary flow situations. The circular shape is important for flood generation, as this kind of cyclone may remain close to a particular river basin in the study region over an extended period and therefore have a high potential for triggering long‐duration rainfall events.

#### 3.1.2 Upper level circulation patterns

**Figure 5:**
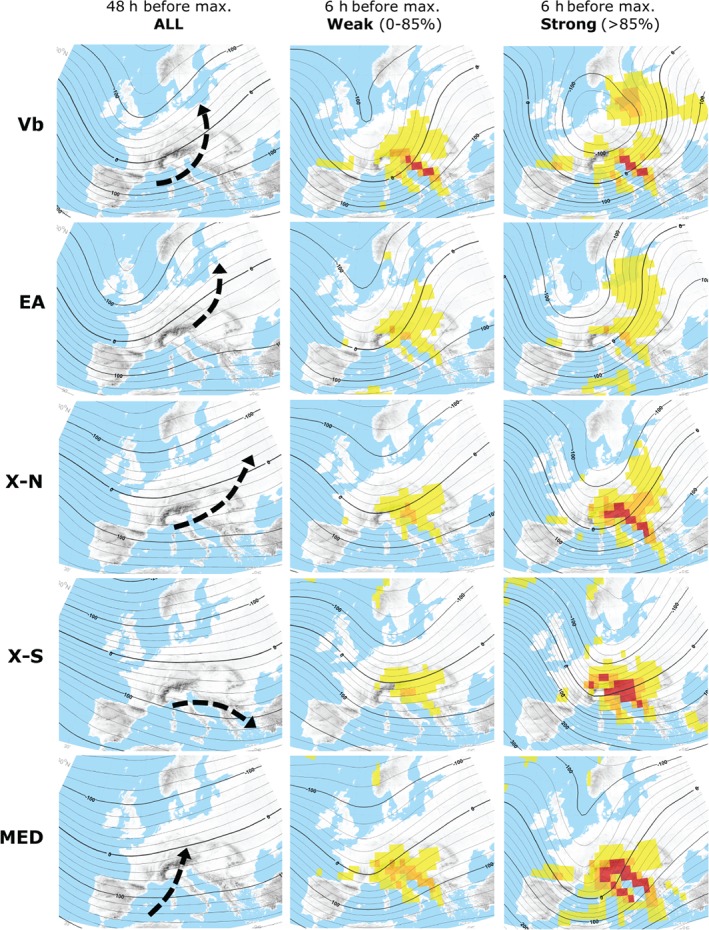
Mean upper level circulation patterns for the track types, 48 h (left column) and 6 h (centre and right column) before the time t_max_ the respective cyclone at SLP reaches its maximum intensity within central Europe (500 hPa geopotential height anomaly). Colour shading indicates mean vertical velocity at time t_max_. −0.3 to −0.6 m s^−1^ shown in yellow, −0.6 to −0.9 m s^−1^ in orange and less than −0.9 m s^−1^ in red. Arrows indicate the typical propagation directions of cyclones at SLP. Type Vb, EA, X‐N, X‐S and MED.

**Figure 6:**
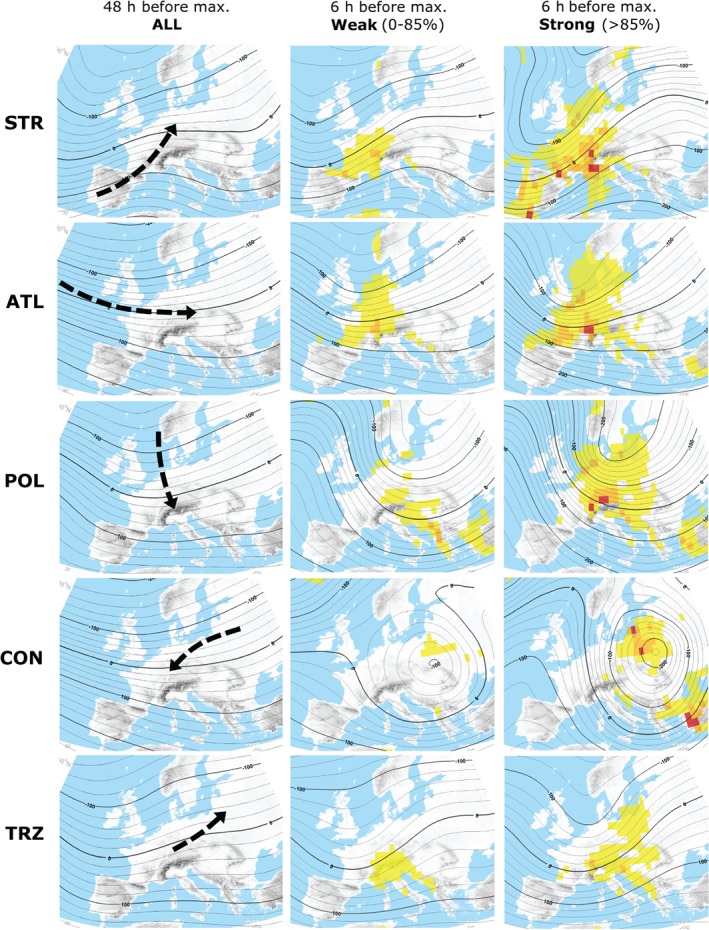
Same as Figure 5 but for types ATL, STR, POL, CON and TRZ.

Precipitation patterns over Europe are largely controlled by atmospheric cyclones, which are embedded in the general circulation of mid latitudes. In the following, dominant patterns of the steering upper level atmospheric circulation over Europe are illustrated in the context of track types identified at SLP. In the left columns of Figures 5 and 6, mean geopotential height anomalies at 500 hPa are shown, 48 h (*t*_−48_) before the corresponding cyclone has reached its maximum intensity inside region TRZ, reflecting the antecedent state of the circulation. The black arrows indicate the typical propagation direction of cyclones at SLP. The centre and left columns show the anomaly fields for weak and strong cyclones, 6 h before the time the cyclones reach their maximum intensity over central Europe (*t*_−6_). The colour shading indicates the mean vertical velocity at Z700 at time *t*_0_. From these figures it becomes clear that each cyclone track type is connected to a specific pattern of the upper level atmospheric flow.

In general, the patterns are characterized by a major trough located over Europe (Figures 5 and 6, left); however, the shape, amplitude and position of the trough differ substantially between the types. The axis of the major trough is located considerably eastwards of central Europe in case of types POL and CON, whereas it is located over western Europe at time *t*_−48_ for all other types. As a consequence, the geostrophic wind vector at 500 hPa has a strong northerly component towards the Alps for these two types, followed by X‐S and ATL with a dominant northwesterly flow, X‐N, MED, STR and TRZ with mostly westerly, and finally Vb and EA with prevailing southwesterly wind over the Alps during the initial phase. The amplitude of the dominant trough also differs between the types. For Vb, EA, POL, STR and CON cyclones the trough is well developed and has a large amplitude which leads to a strong meridional mass and energy exchange over Europe. In contrast, for X‐S, X‐N, MED, ATL and TRZ cyclones the major trough is more elongated with a strong zonal flow over central Europe.

Later in the development (Figures 5 and 6, centre and right), the major trough has propagated further and is located right over central Europe. The exception is STR where the trough is found over the western European coastlines. At the same time the trough has amplified and large regions with enhanced vertical lifting can be seen (yellow colours). Specifically for strong cyclones, these regions are more widespread and contain a number of spots where vertical velocities are further enhanced (red colours). When comparing upper level circulation patterns between weak and strong cyclones, the amplitude of the major trough is even larger for the latter, accompanied by stronger gradients and hence geostrophic winds over Europe. Another prominent feature of the strong Vb, EA and CON‐type cyclones is a major COL that emerges over central Europe, the North Sea and eastern Europe, respectively. Especially for types Vb and CON the COL is very pronounced and therefore prevails over a sizeable region together with circular upper level steering winds for a long time. In 15% of all cases, SLP cyclones of types Vb, EA or X‐N are accompanied by TRZ cyclones at Z700, indicating the occasional presence of upper level COL situations also for the other types.

### 3.2 Precipitation stratified by track types

Both cyclone tracks identified from SLP and Z700 geopotential height are used for the analysis of precipitation with respect to cyclone track types in the following.

#### 3.2.1 Mean cyclone precipitation

Between 57 and 73% of the observed annual precipitation over central Europe can be associated with the occurrence of atmospheric cyclones on average (Table 5), denoted as cyclone precipitation. These numbers are very similar to the findings from other studies for central Europe (Hawcroft *et al*., 2012; Rulfová and Kyselý, 2013) and are about 5% higher than those of Hofstätter *et al*. (2016). The percentage in the current study is largest in areas located around the Eastern Alps close to the Adriatic Sea and decreases towards western Germany. When higher precipitation amounts (>95pct of *R* ^6^) are considered, the fraction increases in all regions to values between 63 and 92%. The attribution of large‐scale precipitation to cyclones therefore appears to be more conclusive for higher precipitation amounts. Relatively low shares are found in region N‐WE and M‐WE, which are most affected by ATL cyclones (Hofstätter *et al*., 2016), but they are occasionally missed when passing by far outside of region TRZ.

**Table 5:** Size of the eight study regions, total annual precipitation and the percentage attributed to cyclones, and mean precipitation in case a cyclone is located within region TRZ.

Region		N‐WE	M‐WE	S‐WE	ALPN	ERZG	N‐CZ	EAST	ALPS
Size of region (10^3^ km^2^)		83.5	26.0	77.8	49.2	48.7	39.6	50.3	34.8
Annual precipitation based on *R* ^6^ (mm year^−1^)		835	756	1140	1216	628	635	678	1098
Percentage attributed to cyclones	*R* ^6^ > 0 mm	57%	62%	67%	65%	64%	66%	69%	73%
	*R* ^6^ > 95pct	63%	76%	83%	81%	86%	91%	90%	92%

**Figure 7:**
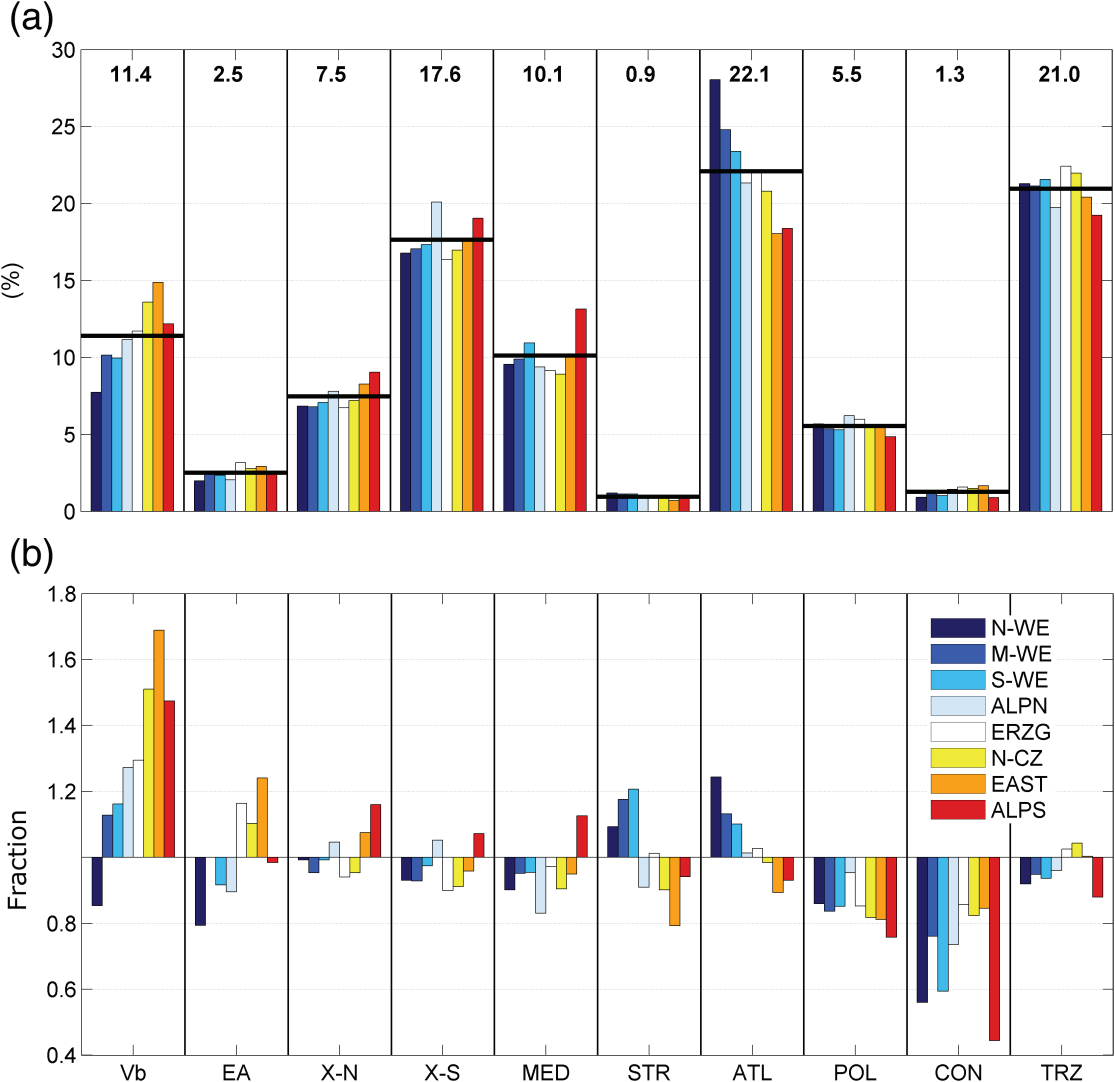
(a) Attribution of mean annual precipitation (based on R ^6^) associated with cyclones to track types (SLP and Z700 together) for the period 1959–2006 as percentages. Black horizontal lines and numbers on top indicate the average over all regions. (b) Regional mean precipitation intensity $\bar{{R^{6}}_{\tau }}$ for each track type τ relative to regional mean precipitation intensity for all track types $\bar{R^{6}}$.

In Figure 7(a) mean annual cyclone precipitation was partitioned into track types for all regions. On an annual basis about 60% is associated with the types TRZ, ATL and X‐S in accordance with the relative frequency of 65% for the occurrence of these track types taken together. Nearly 11% is attributable to Vb cyclones, although their relative frequency is only 5%. In a similar vein, an above average contribution of 7.4% can be seen for type X‐N, a congeneric track type to Vb, and 3% for type EA (consistent with 5.5 and 2% relative frequencies of occurrence). One fifth is attributable to Atlantic cyclones (ATL), another fourth to cyclones emerging straight over central Europe (TRZ) and about one half is connected to cyclones moving from the Mediterranean into central Europe (MED, X‐S, X‐N and Vb). These cyclone types appear as the main drivers of the central European precipitation climate in terms of large‐scale precipitation. The relative contribution from track types also depends on the region (Figure 7(a)). Type ATL, for example, contributes 28% of the annual cyclone precipitation over western Germany but only 18% in the regions east and south of the Eastern Alps (EAST and ALPS). The influence of the Atlantic clearly diminishes from the northwest to the southeast over central Europe. At the same time, the influence of the Mediterranean increases from northwest (41%) to south (53%) regarding type MED, Vb, X‐S and X‐N on an annual basis. Cyclones of types CON, EA, STR and POL only play a minor role in terms of mean annual cyclone precipitation over central Europe. Total annual precipitation in Figure 7(a) is largely driven by cyclone frequency, as the relative share for each type (numbers at the top of Figure 7(a)) is very similar to the relative track frequency (Table 3). However, for some types, such as Vb or CON, the annual totals cannot be explained by frequency alone. Figure 7(b) shows the relative precipitation intensity for each track type, with values larger or smaller than unity indicating above or below average intensities over a certain region. Very high intensities occur for Vb cyclones, especially in regions ALPS, EAST and N‐CZ, which are located close to the path of Vb cyclones. Below average intensities occur for POL and CON cyclones in all regions. STR and ATL cyclones show higher intensities in the western regions.

**Figure 8:**
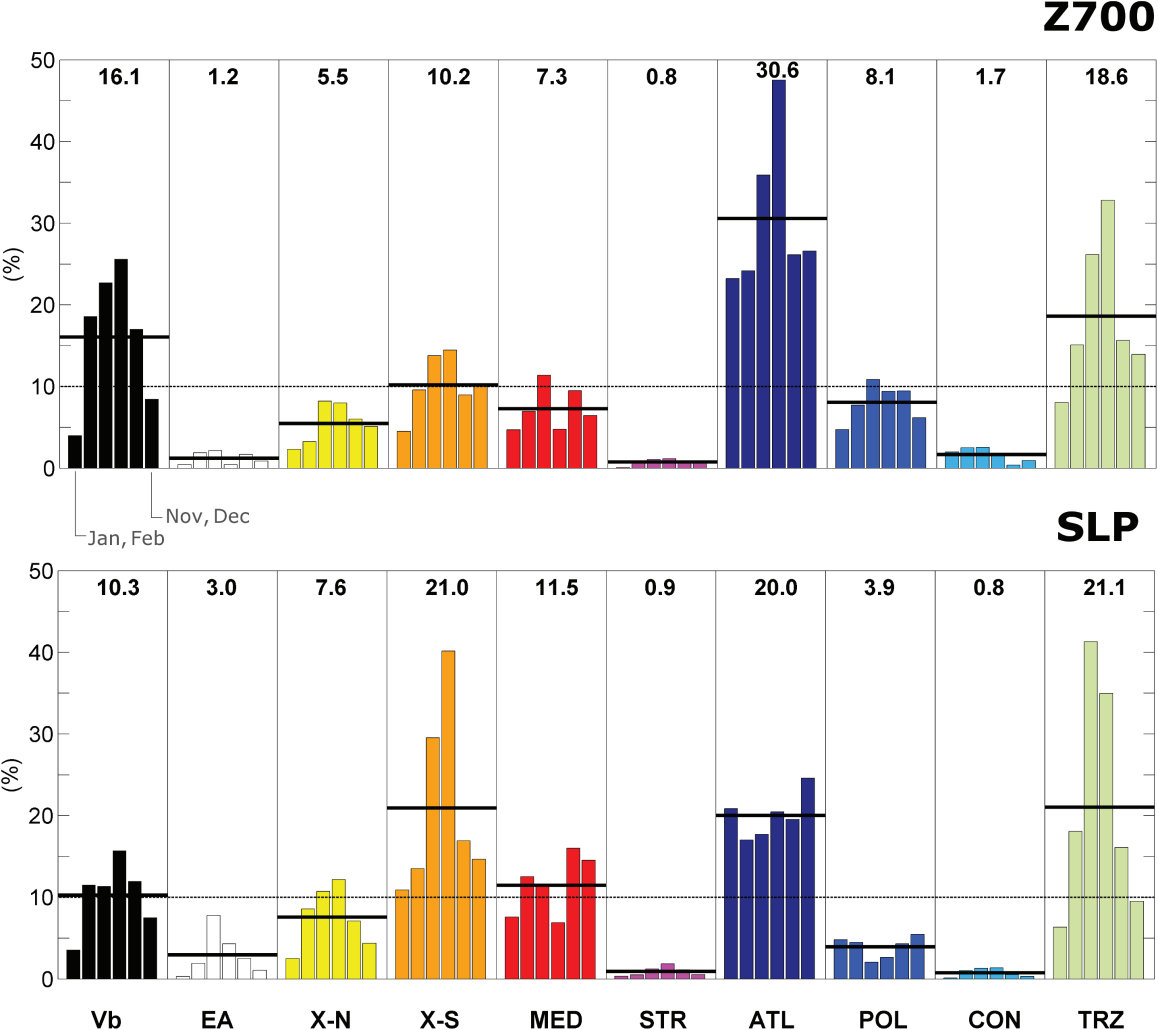
Mean annual cycle of precipitation (based on R ^6^) associated with cyclone tracks for 2‐month periods (bars) as an average contribution over all regions relative to all cyclone types. Black horizontal lines indicate the mean for each track type. (top) Geopotential height at 700 hPa. (bottom) Surface level. Black horizontal lines and numbers on top indicate the average over all seasons.

While Figure 7 represents the mean annual contributions and fractions for each region, Figure 8 represents the seasonal cycle, averaged over all eight regions. The total relative contribution from all bimonthly periods sums up to 1 for each cyclone track type. Similar to Figure 7(a), the relative contribution from the different levels for a certain track type (numbers at the top of Figure 8) corresponds with the ratio of cyclone frequency between SLP and Z700. A clear summer peak in frequency is found for most of the types at both levels. This peak is driven by high temperatures and associated high levels of atmospheric water content during the warm season. An inverse seasonal cycle with a winter maximum is found for ATL and POL at SLP which corresponds with the very high frequency of strong cyclones in winter for these types (Figure 4). Interestingly, there are two maxima of mean precipitation associated with MED cyclones. The first occurs in spring, the second in late autumn, two periods with very different Mediterranean sea surface temperatures. The high frequency of Mediterranean cyclones in March–April and November–December found in this study (Figure 4) is consistent with findings from the literature (Lionello *et al*., 2016). However, the second precipitation maximum already starts in September–October, indicating that additional factors are needed to fully understand the spatio‐temporal characteristics of mean cyclone precipitation over central Europe, apart from cyclone frequency and intensity.

#### 3.2.2 Heavy cyclone precipitation

In this study, heavy precipitation is based on *R* ^trc^ and has been defined as precipitation HP_95_ of a cyclone exceeded by 5% of the cyclones of the same type (Equation (1) and Equation (2)). HP_95_ was calculated for each region and track type separately as well as for all tracks without differentiating between the types. The latter case is used as a reference value to estimate exceedance probabilities and will be denoted as HP_95r_. The extreme value analysis was based on cyclone tracks identified at both atmospheric levels individually, because of important differences in the results. In general, winter heavy precipitation (HP_95r_) is only about 60% of that observed in summer (Table 6), independently of the level. When differentiating between track types, summer HP_95_ is about 10% higher at SLP compared to Z700 for types Vb, EA, X‐N, X‐S, MED and CON, whereas it is about 20% higher at Z700 compared to SLP for types ATL, POL, TRZ and STR. In winter there are no major differences between track types from the two levels. Very high amounts of HP_95_ are observed in the specific case of Vb in both seasons at all levels, with heavy precipitation values between 140 and 190% of the reference. Above or close to average conditions are also found for track types X‐N in both seasons, for EA, X‐S and MED in summer as well as for ATL in winter.

**Table 6:** Heavy precipitation HP_95_ (mm) exceeded by 5% of the cyclone events of the same track type estimated from R ^trc^ as well as reference value HP_95r_ calculated from all tracks (regional means over all regions).

Season	Level	Vb	EA	X‐N	X‐S	MED	STR	ATL	POL	CON	TRZ	HP_95r_
Summer	SLP	56.4	29.9	34.0	30.7	29.7	21.3	24.8	23.6	21.6	25.9	29.9
	700	53.9	21.2	33.6	25.4	28.2	–	30.6	28.5	17.0	28.8	32.4
Winter	SLP	28.9	15.4	20.0	16.3	18.7	14.8	19.7	14.1	14.2	16.1	18.8
	700	25.7	13.3	15.7	14.6	15.2	–	19.8	14.1	12.2	16.2	18.1

Summer: May–October; winter: November–April.

**Figure 9:**
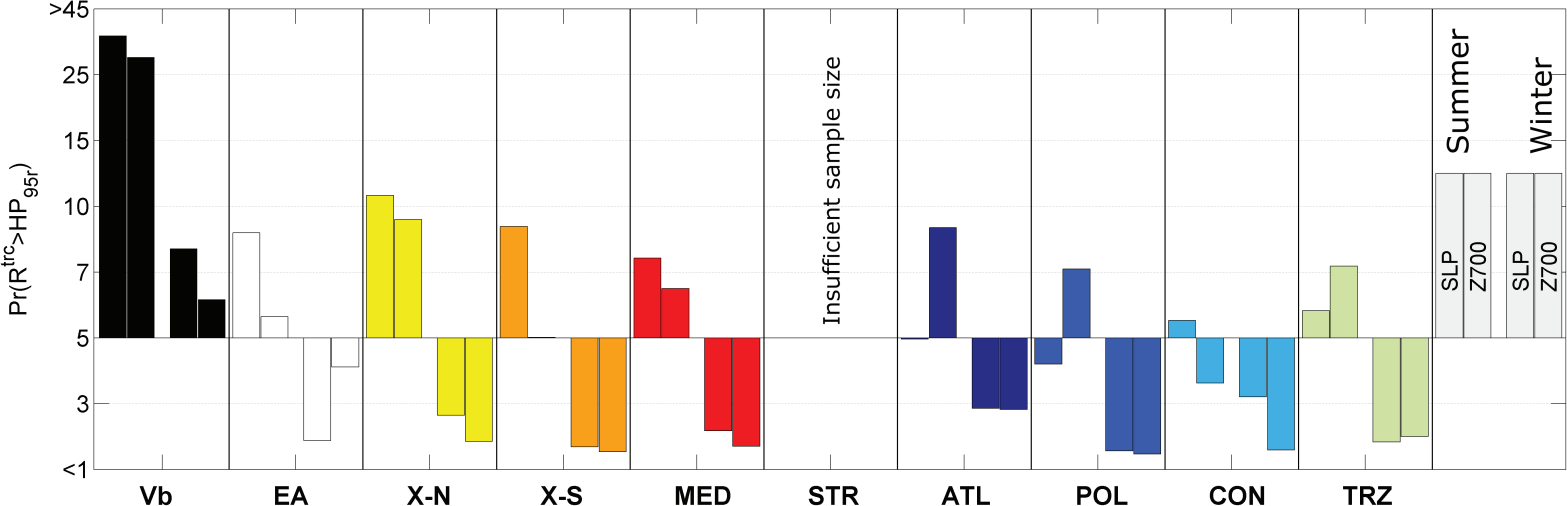
Exceedance probability Pr(R ^trc^ > HP_95r_) for a heavy precipitation event related to the occurrence of cyclones as an average over all regions.

In connection with the seasonal reference values shown in Table 6 (right), the probability Pr(*R* ^trc^ > HP_95r_) of a precipitation event *R* ^trc^ exceeding the threshold HP_95r_ depends on the track type (Figure 9). Probabilities different from 5% indicate a systematic connection between a certain track type and heavy precipitation. For track types ATL, POL and TRZ the probabilities are clearly higher at level Z700, whereas the opposite is the case for types EA and X‐S. For all other types the differences are small. The obvious differences between the levels point towards the importance of considering more than a single atmospheric level in identifying cyclone tracks in the context of large‐scale heavy precipitation analysis.

In terms of differences between regions (not shown), EAST and N‐CZ give the highest probabilities of Vb and EA precipitation, N‐WE and M‐WE the lowest. Type X‐N and X‐S are most relevant in regions located around the Eastern Alps. Some track types show high values (*x* ^prb^ > 5%) in specific regions, such as MED in region ALPS as well as ATL in region N‐WE. Vb types are highly associated with heavy precipitation in all regions (except N‐WE), or more specifically at least every fifth (*x* ^prb^ = 20%) to third (*x* ^prb^ = 30%) Vb cyclone is related to heavy precipitation in the summer season (Figure 9, leftmost). In regions EAST and N‐CZ, HP_95r_ is exceeded during almost every second Vb summer cyclone (*x* ^prb^ = 45%).

#### 3.2.3 Outstanding precipitation events

##### 3.2.3.1 The period 1959–2006 for the eight regions using the WETRAX data

**Figure 10:**
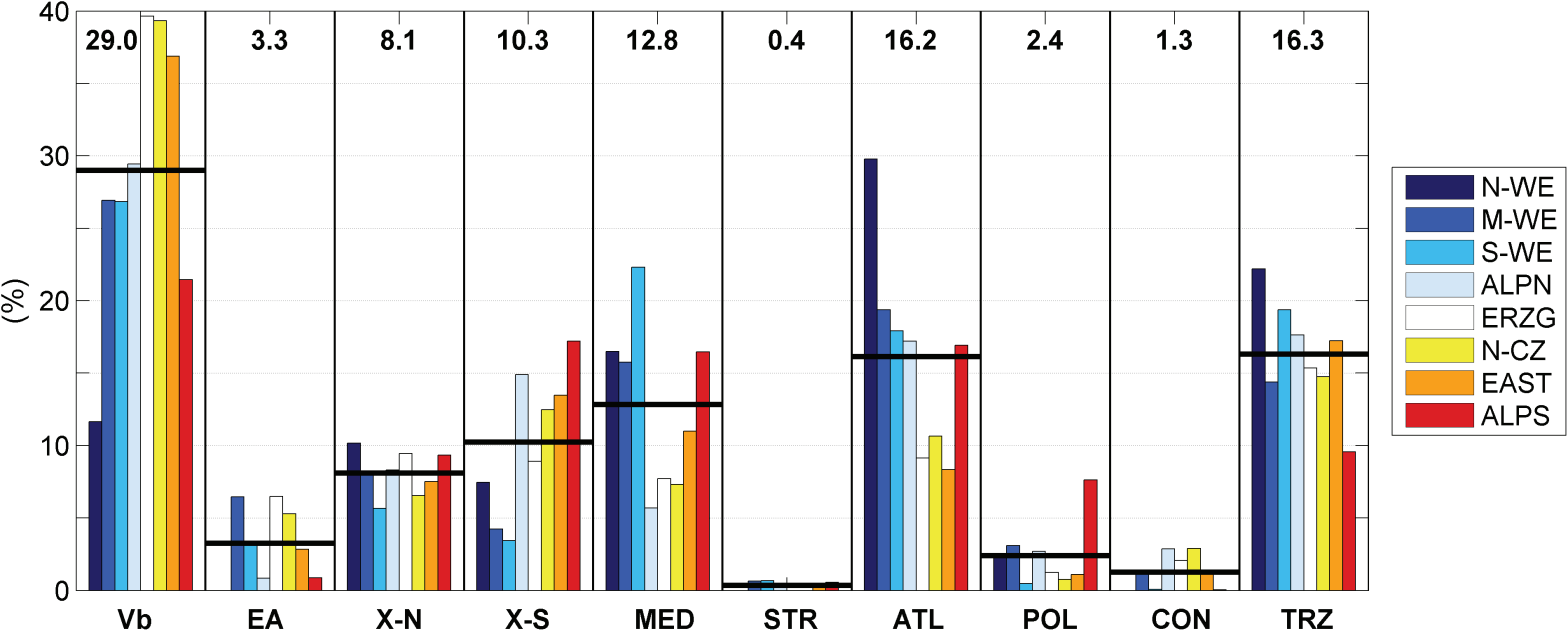
Attribution of the top 50 precipitation events (R ^24^) to track types for 1959–2006 as percentages. Black horizontal lines and numbers on top indicate the average over all regions.

From this analysis (Figure 10), Vb appears as the most frequent cyclone track type among the top 50 precipitation events in central Europe (29% of the events on average over all regions), followed by types TRZ (16%) and ATL (16%), accounting for two thirds of the top events in total. Another third can be attributed to types MED (13%), X‐S (10%) and X‐N (8%). EA appears to be of minor relevance although this type has been related to high HP_95_ exceedance probabilities during summer in Figure 9. This is because this type occurs only rarely. In contrast, ATL and TRZ cyclones are more prominent among the top 50 events than would be expected from the exceedance probabilities. This is mainly because ATL and TRZ cyclones are very frequent, representing about 50% of the total annual frequency which raises the chance of a top event for these types.

The attribution to track types strongly depends on the region, with a large number of ATL and TRZ cyclones dominating the top 50 precipitation events in N‐WE (Figure 10). In the east of the study region between the German Erzgebirge and eastern Austria, Vb cyclones are most important with nearly 40%. Type X‐S on the other hand is more relevant in regions located close to the Adriatic Sea around the Eastern Alps with 18% attribution rate in ALPS, for example. Interestingly, Mediterranean cyclones are also of relevance in this context, especially in regions located near the Western Alps. This finding clearly confirms that subtle spatial differences in the occurrence of heavy precipitation events, even within the limited domain of CE, are caused by different types of atmospheric cyclone tracks.

##### 3.2.3.2 The extended period 1959–2015 for SEGE and CZAT using the E‐OBS data

Table 7 presents a top 50 list of the largest 24 h precipitation events *R* ^24^ of the period 1959–2015. Left shows the results for a domain in SEGE, right the results for a domain located over eastern Austria and the Czech Republic (CZAT). The table contains the rank, the date of the precipitation maximum and the corresponding precipitation amount (*R* ^24^) as well as the attribution to track types from both atmospheric levels using *rdv* ^max^ as described above. The table covers many heavy precipitation events well known from flood history, such as the event 1981–2007 (Rimbu *et al*., 2016), 1977–2007, 1978–2008, 1979–2006 and 1985–2008 (Böhm and Wetzel, 2006), 1977–2008, 1985–2008 and 1997–2007 (Müller *et al*., 2015), 2002–2008 (Ulbrich *et al*., 2003a) and 2013–2016 (Blöschl *et al*., 2013; Schröter *et al*., 2015). For some events, flooding was only minor, even though precipitation was massive, due to dry catchment conditions and/or due to incongruity between precipitation regions and river catchments. Some highly ranked events appear in both regions (e.g. 6–7 July 1985) but also some of the low ranks appear in both lists (e.g. 6–7 August 2010). About 25% of the events are among the top 50 in both regions, SEGE and CZAT, the rest is only in one of them. This indicates that spatio‐temporal coherence, even between neighboring regions, is weak for large‐scale precipitation extremes.

**Table 7:**
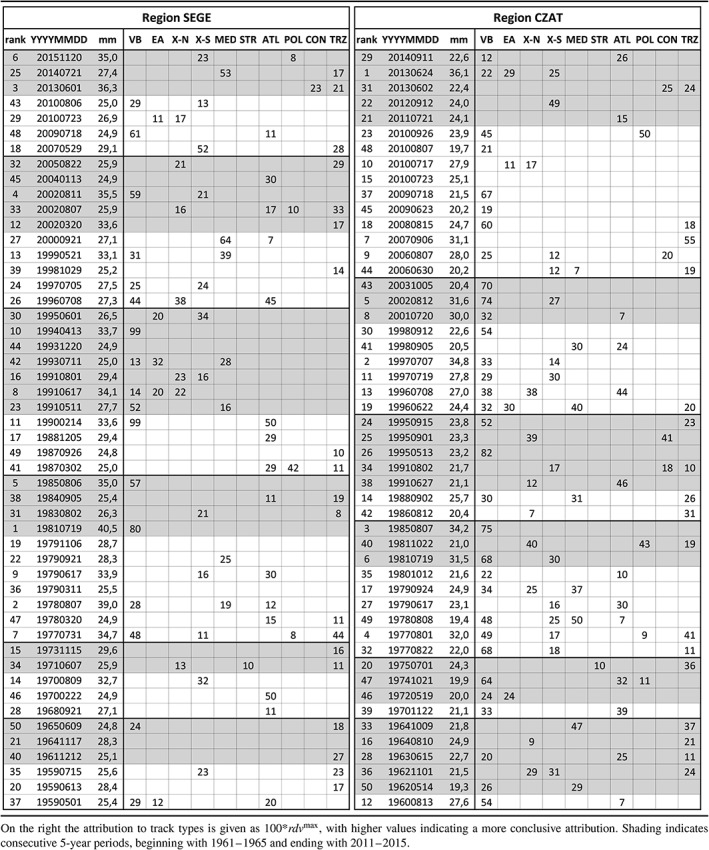
Top 50 of the largest 24 h precipitation maxima (R ^24^) for 1959–2015, with the rank of the events, the corresponding date and the 24 h precipitation amounts on the left.

**Figure 11:**
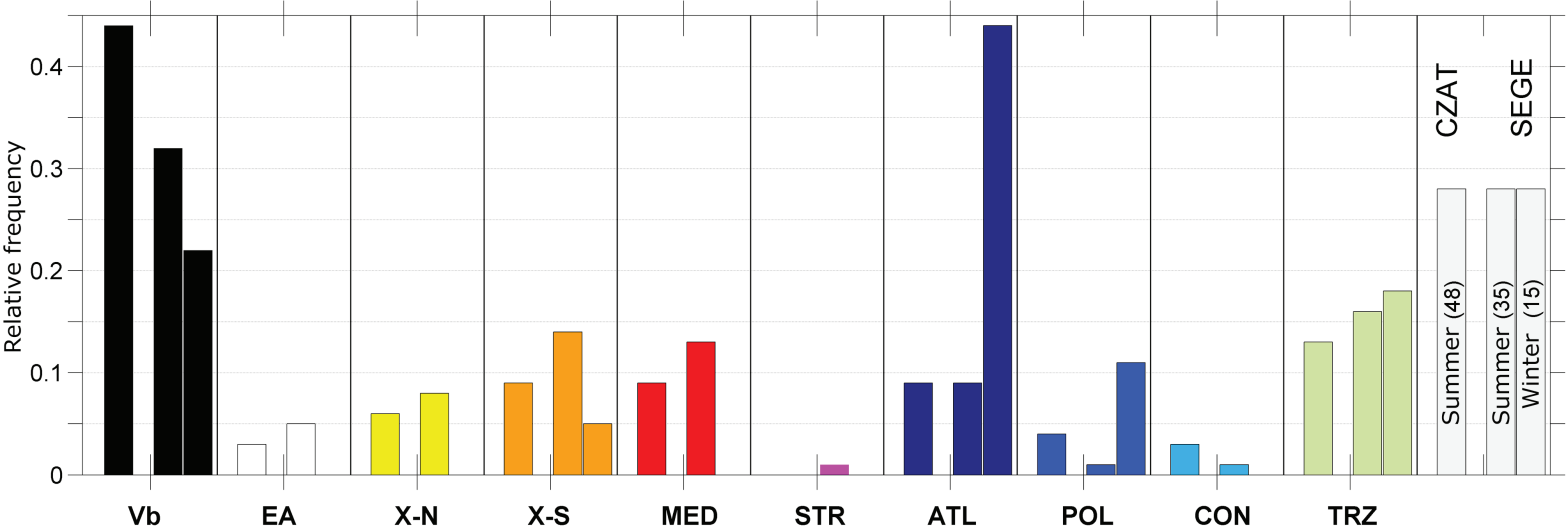
Seasonal relative frequencies of the top 50 precipitation events for the period 1959–2015 in regions CZAT and SEGE. No bars are shown for CZAT in winter with two events only.

Seasonality of the frequency of the top 50 events (Figure 11) shows another important feature. In region CZAT all events occur between May and November, with a distinct frequency peak in August (not shown) when air temperature and sea surface temperatures are highest. In contrast, in SEGE 30% of the top 50 events occur between October and April.

In the cold season (November–April) 44% of the top events found in SEGE can be attributed to ATL cyclones in contrast to only 9% in the warm season (Figure 11). Another 51% can be attributed to Vb (22%), TRZ (18%) and POL (11%) in the cold season. There is also a striking difference between the two regions, although they are located just next to each other. In SEGE cyclone track types, such as ATL, come into play in the cold season, hence a seasonal differentiation is important for understanding central Europe flood regimes from an atmospheric perspective. The most important track type for the extreme precipitation events, both in CZAT and SEGE, is Vb with relative frequencies of 43% (CZAT) and 30% (SEGE); other relevant tracks are TRZ, ATL, X‐S and MED.

Finally, the top 50 events are evaluated in terms of temporal changes of event frequency, of 24 h precipitation as well as of cyclone intensity. Linear trends are estimated from the averages of these variables for consecutive 5‐year periods, beginning with 1961–1965 and ending with 2011–2015. Table 8 presents Mann–Kendall's tau, the according *p*‐value, the observed trend magnitude estimated as the Sen slope, the total trend relative to the overall mean as well as the probability *pct*. Probability *pct* indicates the likelihood to observe a trend larger than the observed one given 50 of the largest precipitation maxima from 1961 to 2015. This likelihood has been calculated from 10^4^ bootstrap samples with replacement, by randomly selecting 50 years out of 1959–2015, assigning these to the given top 50 precipitation amounts and sorting the list by date. For each of these random samples, averages of 5‐year consecutive periods were calculated as described above and trend analysis was applied repeatedly. The individual events were assumed to be independent from each other and the year of the events was assumed to be a result of a uniform random process. From this analysis a small and insignificant increase can be seen for region SEGE, both for the number of events (+4.1%) and the amount of precipitation (+6.9%). For CZAT the increase is stronger with 12.9 and 20.5%, and the change in *R* ^24^ is significant (*p* = 0.06). The increase in event number in CZAT is controlled by the exceptional period 2006–2014, comprising an unusually high number of heavy precipitation events, especially in the year 2010. One can therefore not assume that the increase over the last 50 years is monotonic. The bootstrap analysis shows that the chance for getting a trend larger than the observed one is just 1.1% in region CZAT given the observed precipitation amounts. This clearly demonstrates that the period 2006–2014 was an extraordinary one, specifically in terms of heavy precipitation in the Czech Republic. However, it remains unclear whether this period is just a result of a clustering of events by chance (or natural variability) or related to an ongoing regime shift associated with global climate change. Although a comparable signal cannot be seen for the neighboring region SEGE, a series of heavy precipitation and related flood events hit other European countries as well in the last decade.

**Table 8:** Linear trend analysis of the top 50 precipitation events for consecutive 5‐year periods (1961–2015), regarding 24 h precipitation (R ^24^), number of events and intensity of related cyclones for the CZAT and SEGE regions.

		*k*‐tau	*p*	Sen slope (^−10y^)	Total trend (%)	*pct* (%)
CZAT	*R* ^24^	0.45	0.06	1.0 mm	20.5	1.1
	Number of events	0.29	0.23	0.67	12.9	3.6
	Intensity	0.09	0.76	–	–	72.5
SEGE	*R* ^24^	0.24	0.35	0.4 mm	6.9	18.4
	Number of events	0.16	0.52	0.22	4.1	26.3
	Intensity	−0.09	0.76	–	–	74.9

Mann–Kendall's tau, the according *p*‐value, the trend magnitude estimated as the Sen slope, the total trend (difference 2015–1961) relative to the mean as well as the probability *pct* for getting a trend larger than the observed one given observed precipitation amounts.

## 4 Discussion and conclusions

In this study, atmospheric open and closed cyclones over central Europe have been analysed at two atmospheric levels (SLP and Z700) and classified into track types using the scheme of Hofstätter *et al*. (2016). Atmospheric cyclone track types have been systematically linked to observed precipitation over central Europe, to better understand spatial and seasonal characteristics of precipitation in general as well as of heavy precipitation in particular. Central European cyclone precipitation climate can largely be explained by seasonal track‐type frequency and cyclone intensity; however, additional factors are needed to explain the relatively high fraction of annual precipitation in September and November. The influence of Atlantic cyclones clearly diminishes from northwest to southeast, even within the limited domain of central Europe. Conversely, MED cyclones mostly affect southern regions in autumn, when the Mediterranean sea surface temperatures are still high, as well as between December and April, when MED cyclone frequency is highest.

An upper level COL has been identified as a prominent circulation feature of strong Vb or CON cyclones in this study (Figures 5 and 6). For Vb cyclones, the COL is typically located over central Europe while for CON cyclones it is typically located over eastern Europe. The formation of a COL is a consequence of an upper tropospheric Rossby wave breaking (e.g. Nieto *et al*., 2008), inducing a persistent blocking regime and a series of consecutive cyclones at the surface level, as in June 2013 for example (Grams *et al*., 2014). The occurrence of dominant COL's for selected heavy precipitation events has also been recognized in other studies; however, the exact position of the COL differs in relation to the location of the respective study region. For example, for precipitation relevant to the Elbe basin, the COL is located over Northern Italy (Nied *et al*., 2014); for the Balkan floods in 2014 it was located around the Dinaric Alps (Stadtherr *et al*., 2016); and for flood events in the Czech Republic it is typically located over central Europe (Müller *et al*., 2009). For flood events in CE between 1948 and 2002 an upper level COL was identified, developing over western Europe and propagating across the northern Mediterranean into eastern Europe afterwards (Jacobeit *et al*., 2006). The development of an upper level COL ahead with the occurrence of strong Vb cyclones was identified by Messmer *et al*. (2015), suggesting large‐scale atmospheric dynamics as a main driver of heavy precipitation events. The COL patterns found in the current study for Vb and CON tracks induce an anti‐clockwise circular propagation of the surface cyclone, leading to very long residence times (Table 4). Such a persistent COL might also favor a repetition of similar track types within a short time, which can result in soil moisture saturation at the beginning of a subsequent cyclone leading to enhanced flooding (Blöschl *et al*., 2013).

Vb has been identified as the most relevant track type for heavy cyclone precipitation in all seasons and affects large parts of central Europe, regardless of the level at which the respective cyclone is observed. The reasons for this are: (1) strong Vb cyclones typically occur in all seasons (Hofstätter *et al*., 2016), (2) Vb cyclones remain close to central Europe, much longer than other types, because of a circular cyclone track at the surface level and (3) precipitation intensities, on average, are much higher than for all other types. In connection with the very high exceedance probability for a heavy precipitation event (Table 6 and Figure 10), these findings have serious implications for flood risk, as Vb cyclones can wet up soils within a short time, sometimes independently of the initial soil moisture state (Schröter *et al*., 2015; Nied *et al*., 2017). Another major type for heavy cyclone precipitation is ATL, especially in the western parts of central Europe during the winter half year, when strong ATL cyclones are most frequent. This type explains nearly 50% of the top heavy precipitation events in the SEGE region in the cold season. It is typically connected with a strong northwesterly flow against the Alps, with an embedded warm front leading to northern Stau situations (Seibert *et al*., 2007). Types X‐N, X‐S, EA and MED are mainly relevant for regions around the Eastern Alps in the vicinity of the Adriatic Sea and show highest precipitation amounts in late summer and early autumn.

For the analysis of heavy cyclone precipitation *R* ^trc^ has been used, which depends on the residence time of cyclones over CE, so higher residence times may lead to exaggerated accumulation of precipitation. However, an extended analysis (not shown), based on the largest 24 h running precipitation maxima for each cyclone track, gave very similar results as when using *R* ^trc^ in most regions. This implies that cyclones with very high values of *R* ^trc^ also show very high precipitation amounts on shorter timescales. However, the converse is not necessarily the case. Specifically, in regions N‐WE and M‐WE large‐scale precipitation frequently occurs on daily or sub‐daily timescales because of fast propagating frontal systems connected to Atlantic cyclones. This also explains the lower share of precipitation found in the northwesterly regions such as N‐WE or M‐WE (Table 5), those regions that are located closer to the Atlantic Ocean and far off from major orography as compared to the other regions.

The significance of track type Vb for heavy cyclone precipitation is rather high all over CE. It is especially high in eastern Austria and the Czech Republic. This region is located directly along the path of the Vb cyclone centre at SLP, typically an area with strongest pressure gradients and intense frontal lifting (Pfahl and Sprenger, 2016). At the same time, moist air masses are transported cyclonically around the Eastern Alps into CE (Messmer *et al*., 2015), ascending as a reversed warm conveyor belt over the cold sector of the cyclone (Grams *et al*., 2014). This study also revealed that that precipitation associated with Vb cyclones is between 1.4 and1.9 times larger than the 95% quantile of all tracks (Table 6); it is especially high in summer when seasonal air temperatures are highest. The sensitivity experiments of Messmer *et al*. (2017) using a numerical weather forecast model indicated an upper limit of the Vb cyclone intensities, but not for the related precipitation over central Europe, when sea surface temperature in the Mediterranean is increased by several degrees. This supports the finding from our study of a significant increase of 24 h precipitation for the top 50 events in region CZAT from 1961 to 2015, but the intensity of the related cyclones did not change (Table 8). During this time period a significant and rather monotonic increase of summer mean temperature of 1.5°C was observed in the Greater Alpine Region. Although a comparable precipitation signal cannot be seen for the neighboring region SEGE, a series of heavy precipitation and related flood events did hit other European countries in the last decade. Overall, this indicates a potential increase of heavy precipitation in a warmer future climate for CE. Simulations using the ECHAM5 atmospheric global circulation model (Roeckner *et al*., 2003) at 0.75° horizontal resolution show an increase in heavy precipitation of about 17% in the case of Vb‐type cyclones over CE (Volosciuk *et al*., 2016). In line with Messmer *et al*. (2017), the increase was related to increased air moisture levels over the Mediterranean Basin, but no changes of cyclone intensity were found either.

A couple of caveats are in place regarding this analysis.

1. The cyclone track classification is based on a ‘TRZ’, which has a well‐defined geographical boundary. All cyclones moving into this zone are recognized; however, very large and strong Atlantic systems occasionally move by north of region TRZ and a corresponding track type cannot be assigned, although precipitation was observed in parts of central Europe.
2. Another aspect concerns the exceedance probability for a heavy precipitation event based on *R* ^trc^. It is important to note that *R* ^trc^ generally depends on the duration a cyclone remains within region TRZ. For cyclone types with a high residence time such as Vb, higher amounts of precipitation may be accumulated.
3. Complex flow situations, with more than one cyclone found within region TRZ at the same time, either at SLP or Z700, account for 29% of the time steps with significant precipitation (*R* ^6^ ≥ 1 mm). When considering both levels independently, ambiguous cases are more frequent at SLP (27.4%) than at Z700 (15.1%) due to the underlying orography. This fact has also been pointed out by Wernli and Schwierz (2006) in connection with merging and splitting of cyclones. For a unique attribution of precipitation to one specific cyclone, additional information on the actual synoptic situation would be required.
4. Attribution of precipitation to cyclones in this study only depends on the location of the cyclone centre, i.e. whether it is inside or outside region TRZ. This approach does not consider the actual extent or reach of a cyclone, and precipitation that is not causally related to a cyclone may be attributed incorrectly. Pfahl and Wernli (2012) used a different cyclone tracking scheme that considers the actual extent of cyclones (Wernli and Schwierz, 2006). They found a mean radius of cyclones of between 350 km (genesis stage) and 800 km (4 days later). In the current study 78.8% (77.6%) and 95.5% (94.1%) of precipitation (≥1 mm) was attributed to a cyclone located within a distance less than 800 km and 1000 km at SLP (Z700), respectively, which is in line with Pfahl and Wernli (2012). However, when applying the approach of this study to other locations, we recommend a cyclone size assessment depending on the situation, which does not require comprehensive *a priori* knowledge on the typical local synoptic conditions for the definition of region TRZ.
5. The analysis of heavy precipitation (chap. 3.2.b) and of the top 50 events (chap. 3.2.c) is not affected by convective type and/or small‐scale precipitation, as regional averages have been used. Concerning the precipitation climatology (chap. 3.2.a), convective type precipitation is considered to the extent that station density of the WETRAX data set allows.
6. The size of the regions, over which precipitation has been averaged, may influence the results. Precipitation events with a spatial extent considerably smaller than the regions (Table 5) are systematically attenuated and therefore not fully considered. For these reasons, not every historic flood event observed in the Czech Republic for example (Müller *et al*., 2015) could be confirmed from this study (Table 7). However the occurrence of heavy precipitation alone does not necessarily induce a flood event. Other hydrologic factors can play a role such as antecedent soil moisture, snow melt, the presence of a snow cover and the position of the snowfall line (e.g. Merz and Blöschl, 2003), catchment characteristics (e.g. Gaál *et al*., 2012), river morphology and retention areas (e.g. Skublics *et al*., 2016), as well as flood hazard management.

From this study we conclude that cyclones identified at SLP are more strongly related to large‐scale heavy precipitation over central Europe than those at Z700, in particular track types Vb, EA, X‐N, X‐S, MED and CON. This might be related to the presence of major mountain ranges promoting the genesis or intensification of surface cyclones through lee‐cyclogenesis, as stronger large‐scale precipitation is generally observed in more intense cyclones and especially during the cyclone intensification phase (Pfahl and Sprenger, 2016). A higher precipitation amount is also expected for those cyclones that develop right down to the surface and show a strong signal there, as vertical lifting of moisture can be triggered from deeper atmospheric layers. Also, the association of observed precipitation at a certain location with cyclones appears more distinct at SLP. This is because of a marked horizontal displacement of cyclones between Z700 and SLP during the baroclinic development phase, at times when the vertical axis of a cyclone is tilted backwards. At their major state, cyclones at SLP are usually located 300 km ahead of the associated 500 hPa trough (Lim and Simmonds, 2007). However, tracking cyclones at SLP has a number of challenges, both because of potentially deficient SLP patterns over major orography (e.g. Hoskins and Hodges, 2002) and a high number of spurious vorticity systems found at the leeside of mountain ranges under certain flow conditions. Also splitting and merging is more frequent at lower atmospheric levels as this study showed. On the other hand, higher heavy precipitation amounts were found in all seasons at Z700 for track types ATL, POL or TRZ. The geopotential height at 850 hPa therefore appears as a good compromise for tracking cyclones or vorticity features in the context of heavy precipitation.

The findings of this study provide a new perspective on the central European precipitation climate with a strong emphasis on heavy precipitation. The high frequency of strong cyclones was identified as the key factor in explaining seasonality of mean and heavy precipitation at a regional scale within CE. Strong cyclones not only show enhanced vertical lifting, but also move at considerably lower speeds across CE, resulting in longer residence times. These factors favor high moisture conversion rates as well as a prolonged duration of precipitation. However, to fully explain above average contributions of certain track types in summer and late autumn, additional drivers are needed. Air temperature and Mediterranean sea surface temperature are likely candidates. Model simulations do indicate that Mediterranean sea surface warming amplifies central European precipitation extremes associated with Vb cyclones (Volosciuk *et al*., 2016). Future work could therefore focus on these potential drivers, not only in the context of air moisture supply, but as also on thermodynamic drivers of frontogenetic or cyclogenetic processes in the warm season (Aebischer and Schär, 1998). It remains unclear why Vb cyclones are strong in the warm season, and this appears crucial in understanding potential changes of heavy precipitation events under future climates.

Large‐scale heavy precipitation events have been the focus of this study. Future research could focus on different spatial and temporal scales, for example by considering high intensity short duration events. Serial clustering of cyclones appears to be another important issue, as soil moisture can build up during prolonged rain episodes, increasing the risk of flooding for moderate precipitation extremes. Likewise, temporal changes of track‐type recurrence or cyclone frequency should be considered, as wet spell duration has changed in parts of Europe in recent decades (Zolina *et al*., 2013; Zolina, 2014).

The findings of this study are not only hoped to support the hydro‐meteorological community in understanding the occurrence of large‐scale heavy precipitation and related floods from an atmospheric perspective, but could also provide a valuable basis for evaluating climate models over Europe. For example, frequency, intensity, seasonality and temporal variability of cyclone track types of the models could be compared with those of the observations.

## References

[joc5386-bib-0001] Aebischer U , Schär C . 1998 Low‐level potential vorticity and cyclogenesis to the Lee of the Alps. J. Atmos. Sci. 55: 186–207. https://doi.org/10.1175/1520-0469(1998)055%3C0186:LLPVAC%3E2.0.CO;2.

[joc5386-bib-0002] Bayerisches Landesamt für Umwelt (BLU) . 2006. August – Hochwasser 2005 in Südbayern (August 2005 flood in southern Bavaria), Endbericht vom 12. April 2006, Bayerisches Landesamt für Umwelt, München, 49 pp.

[joc5386-bib-0003] Bayrisches Landesamt für Wasserwirtschaft, BLfW (Hrsg.) . 2003 Hochwasser Mai 1999, Gewässerkundliche Beschreibung. Bayerisches Landesamt für Wasserwirtschaft, München, 2003. http://www.lfu.bayern.de/wasser/hw_ereignisse/aktuell/doc/bericht_pfingsten99.pdf (accessed 18 March 2017).

[joc5386-bib-0004] van Bebber WJ . 1891 Die Zugstrassen der barometrischen Minima nach den Bahnenkarten der deutschen Seewarte für den Zeitraum 1875–1890. Meteorol. Z. 8: 361–366.

[joc5386-bib-0005] Blöschl G , Nester T , Komma J , Parajka J , Perdigão RAP . 2013 The June 2013 flood in the Upper Danube basin, and comparisons with the 2002, 1954 and 1899 floods. Hydrol. Earth Syst. Sci. 17: 5197–5212. https://doi.org/10.5194/hess-17-5197-2013.

[joc5386-bib-0006] Blöschl G , Hall J , Parajka J , Perdigão RAP , Merz B , Arheimer B , Aronica GT , Bilibashi A , Bonacci O , Borga M , Canjevac I , Castellarin A , Chirico GB , Claps P , Fiala K , Frolova N , Gorbachova L , Gül A , Hannaford J , Harrigan S , Kireeva M , Kiss A , Kjeldsen TR , Kohnová S , Koskela JJ , Ledvinka O , Macdonald N , Mavrova‐Guirguinova M , Mediero L , Merz R , Molnar P , Montanari A , Murphy C , Osuch M , Ovcharuk V , Radevski I , Rogger M , Salinas JL , Sauquet E , Šraj M , Szolgay J , Viglione A , Volpi E , Wilson D , Zaimi K , Živkovic N . 2017 Changing climate shifts timing of European floods. Science 357(6351): 588–590. https://doi.org/10.1126/science.aan2506. 28798129 10.1126/science.aan2506

[joc5386-bib-0007] Böhm O , Wetzel KF . 2006 Flood history of the Danube tributaries Lech and Isar in the Alpine foreland of Germany. Hydrol. Sci. J. 51(5): 784–798. https://doi.org/10.1623/hysj.51.5.784.

[joc5386-bib-0008] Casanueva A , Rodríguez‐Puebla C , Frías MD , González‐Reviriego N . 2014 Variability of extreme precipitation over Europe and its relationships with teleconnection patterns. Hydrol. Earth Syst. Sci. 18: 709–725. https://doi.org/10.5194/hess-18-709-2014.

[joc5386-bib-0009] Casty C , Wanner H , Luterbacher J , Esper J , Böhm R . 2005 Temperature and precipitation variability in the European Alps since 1500. Int. J. Climatol. 25(14): 1855–1880. https://doi.org/10.1002/joc.1216.

[joc5386-bib-0010] Coles S . 2001 An Introduction to Statistical Modeling of Extreme Values. Springer Series in Statistics Springer: London 208 pp.

[joc5386-bib-0011] Danhelka J , Kubát J . 2009 Flash Floods on the Territory of the Czech Republic in June and July 2009. Ministry of the Environment of the Czech Republic, Czech Hydrometeorological Institute: Praha (in Czech).

[joc5386-bib-0012] Dayan U , Nissen K , Ulbrich U . 2015 Atmospheric conditions inducing extreme precipitation over the eastern and western Mediterranean. Nat. Hazards Earth Syst. Sci. 15: 2525–2544. https://doi.org/10.5194/nhess-15-2525-2015.

[joc5386-bib-0013] Fleig AK , Tallaksen LM , James P , Hisdal H , Stahl K . 2015 Attribution of European precipitation and temperature trends to changes in synoptic circulation. Hydrol. Earth Syst. Sci. 19: 3093–3107. https://doi.org/10.5194/hess-19-3093-2015.

[joc5386-bib-0014] Flocas AA . 1988 Frontal depressions over the Mediterranean Sea and central southern Europe. Méditerranée 66: 43–52.

[joc5386-bib-0090] Freser F , von Storch H . 2005 A spatial two‐dimensional discrete filter for limited‐area‐model evaluation purposes. Mon. Weather Rev. 133: 1774–1786. https://doi.org/10.1175/MWR2939.1.

[joc5386-bib-0015] Gaál L , Szolgay J , Kohnová S , Parajka J , Merz R , Viglione A , Blöschl G . 2012 Flood timescales: understanding the interplay of climate and catchment processes through comparative hydrology. Water Resour. Res. 48: W04511 https://doi.org/10.1029/2011WR011509.

[joc5386-bib-0016] Grams CM , Binder H , Pfahl S , Piaget N , Wernli H . 2014 Atmospheric processes triggering the central European floods in June 2013. Nat. Hazards Earth Syst. Sci. 14: 1691–1702. https://doi.org/10.5194/nhess-14-1691-2014.

[joc5386-bib-0017] Grazzini F , van der Grijn G . 2002 Central European floods during summer 2002. ECMWF Newsl. 96: 18–28.

[joc5386-bib-0018] Hall J , Arheimer B , Borga M , Brázdil R , Claps P , Kiss A , Kjeldsen TR , Kriaučiūnienė J , Kundzewicz ZW , Lang M , Llasat MC , Macdonald N , McIntyre N , Mediero L , Merz B , Merz R , Molnar P , Montanari A , Neuhold C , Parajka J , Perdigão RAP , Plavcová L , Rogger M , Salinas JL , Sauquet E , Schär C , Szolgay J , Viglione A , Blöschl G . 2014 Understanding flood regime changes in Europe: a state‐of‐the‐art assessment. Hydrol. Earth Syst. Sci. 18: 2735–2772. https://doi.org/10.5194/hess-18-2735-2014.

[joc5386-bib-0019] Hantel M (ed). 2005 Observed Global Climate. LandoltBörnstein: Numerical Data and Functional Relationships in Science and Technology, Vol. 6 Springer: Berlin.

[joc5386-bib-0020] Harada Y , Kamahori H , Kobayashi C , Endo H , Kobayashi S , Ota Y , Onoda H , Onogi K , Miyaoka K , Takahashi K . 2016 The JRA‐55 reanalysis: representation of atmospheric circulation and climate variability. J. Meteorol. Soc. Jpn. 94: 269–302. https://doi.org/10.2151/jmsj.2016-015.

[joc5386-bib-0021] Haslinger K , Blöschl G . 2017 Space‐time patterns of meteorological drought events in the European Greater Alpine Region over the past 210 years. Water Resour. Res. 53 https://doi.org/10.1002/2017WR020797.

[joc5386-bib-0022] Hawcroft MK , Shaffrey LC , Hodges KI , Dacre HF . 2012 How much Northern Hemisphere precipitation is associated with extratropical cyclones? Geophys. Res. Lett. 39: L24809 https://doi.org/10.1029/2012GL053866.

[joc5386-bib-0023] Haylock MR , Hofstra N , Klein Tank AMG , Klok EJ , Jones PD , New M . 2008 A European daily high‐resolution gridded data set of surface temperature and precipitation for 1950–2006. J. Geophys. Res. 113: D20119 https://doi.org/10.1029/2008JD010201.

[joc5386-bib-0024] Hodges KI , Lee RW , Bengtsson L . 2011 A comparison of extratropical cyclones in recent reanalyses ERA‐Interim, NASA MERRA, NCEP CFSR, and JRA‐25. J. Clim. 24(18): 4888–4906. https://doi.org/10.1175/2011JCLI4097.1.

[joc5386-bib-0025] Hofstätter M , Chimani B . 2012 van Bebber's cyclone tracks at 700 hPa in the eastern Alps for 1961–2002 and their comparison to circulation type classifications. Meteorol. Z. 21(5): 459–473. https://doi.org/10.1127/0941-2948/2012/0473.

[joc5386-bib-0026] Hofstätter M , Jacobeit J , Homann M , Lexer A , Chimani B , Philipp A , Beck C , Ganekind M . 2015. WETRAX – weather patterns, cyclone tracks and related precipitation extremes. Großflächige Starkniederschläge im Klimawandel in Mitteleuropa, Final Report, Geographica Augustana 19, Universität Augsburg, Augsburg, Germany.

[joc5386-bib-0027] Hofstätter M , Chimani B , Lexer A , Blöschl G . 2016 A new classification scheme of European cyclone tracks with relevance to precipitation. Water Resour. Res. 52: 7086–7104. https://doi.org/10.1002/2016WR019146.

[joc5386-bib-0028] Hofstra N , New M , McSweeney C . 2010 The influence of interpolation and station network density on the distributions and trends of climate variables in gridded daily data. Clim. Dyn. 35(5): 841–858.

[joc5386-bib-0029] Hoskins BJ , Hodges KI . 2002 New perspectives on the Northern Hemisphere winter storm tracks. J. Atmos. Sci. 59: 1041–1061. https://doi.org/10.1175/1520-0469(2002)059%3C1041:NPOTNH%3E2.0.CO;2.

[joc5386-bib-0030] Jacobeit J . 1993 Regionale Unterschiede im atmosphärischen Zirkulationsgeschehen bei globalen Klimaveränderungen. Die Erde 124: 63–77.

[joc5386-bib-0031] Jacobeit J , Philipp A , Nonnenmacher M . 2006 Atmospheric circulation dynamics linked with prominent discharge events in central Europe. Hydrol. Sci. J. 51: 946–965. https://doi.org/10.1623/hysj.51.5.946.

[joc5386-bib-0032] Japanese Meteorological Agency . 2013 JRA‐55: Japanese 55‐year reanalysis, daily 3‐hourly and 6‐hourly data. Computational and Information Systems Laboratory, National Center for Atmospheric Research: Tokyo (accessed 16 May 2016). https://doi.org/10.5065/D6HH6H41.

[joc5386-bib-0033] Kašpar M , Müller M . 2014 Combinations of large‐scale circulation anomalies conducive to precipitation extremes in the Czech Republic. Atmos. Res. 138: 205–212.

[joc5386-bib-0034] Kobayashi S , Ota Y , Harada Y , Ebita A , Moriya M , Onoda H , Onogi K , Kamahori H , Kobayashi C , Endo H , Miyaoka K , Takahashi K . 2015 The JRA‐55 reanalysis: general specifications and basic characteristics. J. Meteorol. Soc. Jpn. 93: 5–48. https://doi.org/10.2151/jmsj.2015-001.

[joc5386-bib-0035] Kottek M , Grieser J , Beck C , Rudolf B , Rubel F . 2006 World map of the Köppen–Geiger climate classification updated. Meteorol. Z. 15: 259–263. https://doi.org/10.1127/0941-2948/2006/0130.

[joc5386-bib-0036] Kundzewicz ZW , Szamalek K , Kowalczak P . 1999 The great flood of 1997 in Poland. Hydrol. Sci. J. 44(6): 855–870.

[joc5386-bib-0037] L'Ecuyer TS , Beaudoing HK , Rodell M , Olson W , Lin B , Kato S , Clayson CA , Wood E , Sheffield J , Adler R , Huffman G , Bosilovich M , Gu G , Robertson F , Houser PR , Chambers D , Famiglietti JS , Fetzer E , Liu WT , Gao X , Schlosser CA , Clark E , Lettenmaier DP , Hilburn K . 2015 The observed state of the energy budget in the early twenty‐first century. J. Clim. 28: 8319–8346. https://doi.org/10.1175/JCLI-D-14-00556.1.

[joc5386-bib-0038] Lim EP , Simmonds I . 2007 Southern Hemisphere winter extratropical cyclone characteristics and vertical organization observed with the ERA‐40 data in 1979–2001. J. Clim. 20: 2675–2690. https://doi.org/10.1175/JCLI4135.1.

[joc5386-bib-0039] Lionello P , Trigo IF , Gil V , Liberato MLR , Nissen K , Pinto JG , Raible CC , Reale M , Tanzarella A , Trigo RM , Ulbrich S , Ulbrich U . 2016 Objective climatology of cyclones in the Mediterranean region: a consensus view among methods with different system identification and tracking criteria. Tellus A 68: 29391 https://doi.org/10.3402/tellusa.v68.29391.

[joc5386-bib-0040] Llasat MC , Puigcerver M . 1990 Cold air pools over Europe. Meteorol. Atmos. Phys. 42: 171–177. https://doi.org/10.1007/BF01314823.

[joc5386-bib-0041] Masson D , Frei C . 2016 Long‐term variations and trends of mesoscale precipitation in the Alps: recalculation and update for 1901–2008. Int. J. Climatol. 36: 492–500. https://doi.org/10.1002/joc.4343.

[joc5386-bib-0042] Merz R , Blöschl G . 2003 A process typology of regional floods. Water Resour. Res. 39(12): 1340 https://doi.org/10.1029/2002WR001952.

[joc5386-bib-0043] Messmer M , Gómez‐Navarro JJ , Raible CC . 2015 Climatology of Vb cyclones, physical mechanisms and their impact on extreme precipitation over central Europe. Earth Syst. Dyn. 6: 541–553. https://doi.org/10.5194/esd-6-541-2015.

[joc5386-bib-0044] Messmer M , Gómez‐Navarro JJ , Raible CC . 2017 Sensitivity experiments on the response of Vb cyclones to sea surface temperature and soil moisture changes. Earth Syst. Dyn. 8: 477–493. https://doi.org/10.5194/esd-8-477-2017.

[joc5386-bib-0045] Müller M , Kašpar M , Matschullat J . 2009 Heavy rains and extreme rainfall‐runoff events in central Europe from 1951 to 2002. Nat. Hazards Earth Syst. Sci. 9: 441–450. https://doi.org/10.5194/nhess-9-441-2009.

[joc5386-bib-0046] Müller M , Kašpar M , Valeriánová A , Crhová L , Holtanová E , Gvoždíková B . 2015 Novel indices for the comparison of precipitation extremes and floods: an example from the Czech territory. Hydrol. Earth Syst. Sci. 19: 4641–4652. https://doi.org/10.5194/hess-19-4641-2015.

[joc5386-bib-0091] Murray R , Simmonds I . 1991 A numerical scheme for tracking cyclone centres from digital data. Part I: development and operation of the scheme. Aust. Meteorol. Mag. 39: 155–166.

[joc5386-bib-0047] Neu U , Akperov MG , Bellenbaum N , Benestad R , Blender R , Caballero R , Cocozza A , Dacre HF , Feng Y , Fraedrich K , Grieger J , Gulev S , Hanley J , Hewson T , Inatsu M , Keay K , Kew SF , Kindem I , Leckebusch GC , Liberato MLR , Lionello P , Mokhov II , Pinto JG , Raible CC , Reale M , Rudeva I , Schuster M , Simmonds I , Sinclair M , Sprenger M , Tilinina ND , Trigo IF , Ulbrich S , Ulbrich U , Wang XL , Wernli H . 2013 IMILAST – a community effort to intercompare extratropical cyclone detection and tracking algorithms. Bull. Am. Meteorol. Soc. 94: 529–547.

[joc5386-bib-0048] Nied M , Pardowitz T , Nissen K , Ulbrich U , Hundecha Y , Merz B . 2014 On the relationship between hydro‐meteorological patterns and flood types. J. Hydrol. 519(Part D): 3249–3262. https://doi.org/10.1016/j.jhydrol.2014.09.089.

[joc5386-bib-0049] Nied M , Schröter K , Lüdtke S , Nguyen D , Merz B . 2017 What are the hydro‐meteorological controls on flood characteristics? J. Hydrol. 545: 310–326. https://doi.org/10.1016/j.jhydrol.2016.12.003.

[joc5386-bib-0092] Nieto R , Sprenger M , Wernli H , Trigo RM , Gimeno L . 2008 Identification and climatology of cut‐off low near the tropopause. Ann. N. Y. Acad. Sci. 1146: 256–290. https://doi.org/10.1196/annals.1446.016. 19076419 10.1196/annals.1446.016

[joc5386-bib-0050] Nissen K , Katrin M , Ulbrich U , Leckebusch G . 2013 Vb cyclones and associated rainfall extremes over central Europe under present day and climate change conditions. Meteorol. Z. 22(6): 649–660. https://doi.org/10.1127/0941-2948/2013/0514.

[joc5386-bib-0051] Panofsky HA , Brier GW . 1968 Some Applications of Statistics to Meteorology. Pennsylvania State University Press: University Park, PA, 40–45.

[joc5386-bib-0052] Parajka J , Kohnová S , Bálint G , Barbuc M , Borga M , Claps P , Cheval S , Dumitrescu A , Gaume E , Hlavová K , Merz R , Pfaundler M , Stancalie G , Szolgay J , Blöschl G . 2010 Seasonal characteristics of flood regimes across the Alpine–Carpathian range. J. Hydrol. 394: 78–89. https://doi.org/10.1016/j.jhydrol.2010.05.015. 10.1016/j.jhydrol.2010.05.015 PMC4106690 25067854

[joc5386-bib-0053] Pauling A , Luterbacher J , Casty C , Wanner H . 2006 500 years of gridded high‐resolution precipitation reconstructions over Europe and the connection to large‐scale circulation. Clim. Dyn. 26: 387–405.

[joc5386-bib-0054] Peel MC , McMahon TA , Finlayson BL . 2004 Continental differences in the variability of annual runoff – update and reassessment. J. Hydrol. 295: 185–197. https://doi.org/10.1016/j.jhydrol.2004.03.004.

[joc5386-bib-0055] Pfahl S , Sprenger M . 2016 On the relationship between extratropical cyclone precipitation and intensity. Geophys. Res. Lett. 43: 1752–1758. https://doi.org/10.1002/2016GL068018.

[joc5386-bib-0056] Pfahl S , Wernli H . 2012 Quantifying the relevance of cyclones for precipitation extremes. J. Clim. 25: 6770–6780. https://doi.org/10.1175/JCLI-D-11-00705.1.

[joc5386-bib-0093] Pinto JG , Spangehl T , Ulbrich U , Speth P . 2005 Sensitivities of a cyclone detection and tracking algorithm: individual tracks and climatology. Meteorol. Z. 14: 823–838. https://doi.org/10.1127/0941-2948/2005/0068.

[joc5386-bib-0057] Raible CC , Della‐Marta P , Schwierz C , Wernli H , Blender R . 2008 Northern Hemisphere extratropical cyclones: a comparison of detection and tracking methods and different reanalyses. Mon. Weather Rev. 136(3): 880–897. https://doi.org/10.1175/2007MWR2143.1.

[joc5386-bib-0058] Rauthe M , Steiner H , Riediger U , Mazurkiewicz A , Gratzki A . 2013 A central European precipitation climatology – Part I: generation and validation of a high‐resolution gridded daily data set (HYRAS). Meteorol. Z. 22: 235–256. https://doi.org/10.1127/0941-2948/2013/0436.

[joc5386-bib-0059] Richman MB . 1986 Rotation of principal components. Int. J. Climatol. 6: 293–335. https://doi.org/10.1002/joc.3370060305.

[joc5386-bib-0060] Rimbu N , Treut HL , Janicot S , Boroneant C , Laurent C . 2001 Decadal precipitation variability over Europe and its relation with surface atmospheric circulation and sea surface temperature. Q. J. R. Meteorol. Soc. 127: 315–329. https://doi.org/10.1002/qj.49712757204.

[joc5386-bib-0061] Rimbu N , Czymzik M , Ionita M , Lohmann G , Brauer A . 2016 Atmospheric circulation patterns associated with the variability of River Ammer floods: evidence from observed and proxy data. Clim. Past 12: 377–385. https://doi.org/10.5194/cp-12-377-2016.

[joc5386-bib-0062] Roeckner E , Bäuml G , Bonaventura L , Brokopf R , Esch M , Giorgetta M , Hagemann S , Kirchner I , Kornblueh L , Manzini E , Rhodin A , Schlese U , Schulzweida U , Tompkins A . 2003. The atmospheric general circulation model ECHAM5, part 1, model description. Report No. 349, Max‐Planck Institute for Meteorology, Hamburg, Germany, p. 127.

[joc5386-bib-0063] Rubel F , Kottek M . 2010 Observed and projected climate shifts 1901–2100 depicted by world maps of the Köppen–Geiger climate classification. Meteorol. Z. 19: 135–141. https://doi.org/10.1127/0941-2948/2010/0430.

[joc5386-bib-0064] Rulfová Z , Kyselý J . 2013 Disaggregating convective and stratiform precipitation from station weather data. Atmos. Res. 134: 100–115. https://doi.org/10.1016/j.atmosres.2013.07.015.

[joc5386-bib-0066] Scherrer SC , Begert M , Croci‐Maspoli M , Appenzeller C . 2016 Long series of Swiss seasonal precipitation: regionalization, trends and influence of large‐scale flow. Int. J. Climatol. 36: 3673–3689. https://doi.org/10.1002/joc.4584.

[joc5386-bib-0067] Schmidli J , Schmutz C , Frei C , Wanner H , Schär C . 2002 Mesoscale precipitation variability in the region of the European Alps during the 20th century. Int. J. Climatol. 22: 1049–1074. https://doi.org/10.1002/joc.769.

[joc5386-bib-0068] Schneidereit A , Blender R , Fraedrich K . 2010 A radius‐depth model for midlatitude cyclones in reanalysis data and simulations. Q. J. R. Meteorol. Soc. 136(646): 50–60. https://doi.org/10.1002/qj.523.

[joc5386-bib-0069] Schröter K , Kunz M , Elmer F , Mühr B , Merz B . 2015 What made the June 2013 flood in Germany an exceptional event? A hydro‐meteorological evaluation. Hydrol. Earth Syst. Sci. 19: 309–327. https://doi.org/10.5194/hess-19-309-2015.

[joc5386-bib-0070] Seibert P , Frank A , Formayer H . 2007 Synoptic and regional patterns of heavy precipitation in Austria. Theor. Appl. Climatol. 87: 139–153. https://doi.org/10.1007/s00704-006-0198-8.

[joc5386-bib-0094] Simmonds I , Murray RJ , Leighton RM . 1999 A refinement cyclone tracking methods with data from FROST. Aust. Meteorol. Mag. Special edition, 35–49.

[joc5386-bib-0071] Skublics D , Blöschl G , Rutschmann P . 2016 Effect of river training on flood retention of the Bavarian Danube. J. Hydrol. Hydromech. 64(4): 349–356. https://doi.org/10.1515/johh-2016-0035.

[joc5386-bib-0072] Stadtherr L , Coumou D , Petoukhov V , Petri S , Rahmstorf S . 2016 Record Balkan floods of 2014 linked to planetary wave resonance. Sci. Adv. 2(4): e1501428 https://doi.org/10.1126/sciadv.1501428. 27152340 10.1126/sciadv.1501428 PMC4846427

[joc5386-bib-0073] Stucki P , Rickli R , Brönnimann S , Martius O , Wanner H , Grebner D , Luterbacher J . 2012 Weather patterns and hydro‐climatological precursors of extreme floods in Switzerland since 1868. Meteorol. Z. 21(6): 531–550. https://doi.org/10.1127/0941-2948/2012/36.

[joc5386-bib-0074] Themeßl MJ , Gobiet A , Leuprecht A . 2011 Empirical‐statistical downscaling and error correction of daily precipitation from regional climate models. Int. J. Climatol. 31: 1530–1544. https://doi.org/10.1002/joc.2168.

[joc5386-bib-0075] Tilinina N , Gulev SK , Rudeva I , Koltermann P . 2013 Comparing cyclone life cycle characteristics and their interannual variability in different reanalyses. J. Clim. 26: 6419–6438. https://doi.org/10.1175/JCLI-D-12-00777.1.

[joc5386-bib-0076] Trenberth KE , Stepaniak DP . 2004 The flow of energy through the Earth's climate system. Q. J. R. Meteorol. Soc. 130: 2677–2701. https://doi.org/10.1256/qj.04.83.

[joc5386-bib-0077] Trenberth KE , Dai A , Rasmussen RM , Parsons DB . 2003 The changing character of precipitation. Bull. Am. Meteorol. Soc. 84: 1205–1217. https://doi.org/10.1175/BAMS-84-9-1205.

[joc5386-bib-0078] Ulbrich U , Brücher T , Fink AH , Leckebusch GC , Krüger A , Pinto JG . 2003a The central European floods in August 2002, Part I: rainfall periods and flood development. Weather 58: 371–376.

[joc5386-bib-0079] Ulbrich U , Brücher T , Fink AH , Leckebusch GC , Krüger A , Pinto JG . 2003b The central European floods in August 2002, Part II: synoptic causes and considerations with respect to climatic change. Weather 58: 434–441.

[joc5386-bib-0080] Uppala SM , KÅllberg PW , Simmons AJ , Andrae U , Bechtold VDC , Fiorino M , Gibson JK , Haseler J , Hernandez A , Kelly GA , Li X , Onogi K , Saarinen S , Sokka N , Allan RP , Andersson E , Arpe K , Balmaseda MA , Beljaars ACM , Berg LVD , Bidlot J , Bormann N , Caires S , Chevallier F , Dethof A , Dragosavac M , Fisher M , Fuentes M , Hagemann S , Hólm E , Hoskins BJ , Isaksen L , Janssen PAEM , Jenne R , Mcnally AP , Mahfouf JF , Morcrette JJ , Rayner NA , Saunders RW , Simon P , Sterl A , Trenberth KE , Untch A , Vasiljevic D , Viterbo P , Woollen J . 2005 The ERA‐40 re‐analysis. Q. J. R. Meteorol. Soc. 131: 2961–3012. https://doi.org/10.1256/qj.04.176.

[joc5386-bib-0081] Volosciuk C , Maraun D , Semenov VA , Tilinina N , Gulev SK , Latif M . 2016 Rising Mediterranean sea surface temperatures amplify extreme summer precipitation in central Europe. Sci. Rep. 6: 32450 https://doi.org/10.1038/srep32450. 27573802 10.1038/srep32450 PMC5004138

[joc5386-bib-0082] Wernli H , Schwierz C . 2006 Surface cyclones in the ERA‐40 dataset, part I, novel identification method and global climatology. J. Atmos. Sci. 63: 2486–2507.

[joc5386-bib-0083] Westra S , Fowler HJ , Evans JP , Alexander LV , Berg P , Johnson F , Kendon EJ , Lenderink G , Roberts NM . 2014 Future changes to the intensity and frequency of short‐duration extreme rainfall. Rev. Geophys. 52: 522–555. https://doi.org/10.1002/2014RG000464.

[joc5386-bib-0084] Zolina O . 2014 Multidecadal trends in the duration of wet spells and associated intensity of precipitation as revealed by very dense observational network. Environ. Res. Lett. 9: 025003 https://doi.org/10.1088/1748-9326/9/2/025003.

[joc5386-bib-0085] Zolina O , Simmer C , Belyaev K , Gulev SK , Koltermann P . 2013 Changes in the duration of European wet and dry spells during the last 60 years. J. Clim. 26(6): 2022–2047. https://doi.org/10.1175/JCLI-D-11-00498.1.

[joc5386-bib-0086] Zolina O , Simmer C , Kapala A , Shabanov P , Becker P , Maechel H , Gulev SK , Groisman P . 2014 Precipitation variability and extremes in central Europe: new view from STAMMEX results. Bull. Am. Meteorol. Soc. 95: 995–1002.

[joc5386-bib-0087] Zveryaev II . 2004 Seasonality in precipitation variability over Europe. J. Geophys. Res. 109: D05103 https://doi.org/10.1029/2003JD003668.

[joc5386-bib-0088] Zveryaev II . 2006 Seasonally varying modes in long‐term variability of European precipitation during the 20th century. J. Geophys. Res. 111: D21116 https://doi.org/10.1029/2005JD006821.

[joc5386-bib-0089] Zveryaev II , Allan RP . 2010 Summertime precipitation variability over Europe and its links to atmospheric dynamics and evaporation. J. Geophys. Res. 115: D12102 https://doi.org/10.1029/2008JD011213.

